# Anxiety at age 15 predicts psychiatric diagnoses and suicidal ideation in late adolescence and young adulthood: results from two longitudinal studies

**DOI:** 10.1186/s12888-019-2349-3

**Published:** 2019-11-14

**Authors:** Sabrina Doering, Paul Lichtenstein, Christopher Gillberg, NTR, D. I. Boomsma, Toos C. E. M. van Beijsterveldt, Lannie Ligthart, Gonneke Willemsen, Eco de Geus, Christel M. Middeldorp, Meike Bartels, Ralf Kuja-Halkola, Sebastian Lundström

**Affiliations:** 1 Centre for Ethics, Law and Mental Health, University of Gothenburg, Gothenburg, Sweden; 2 Department of Medical Epidemiology and Biostatics, Karolinska Institutet, Stockholm, Sweden; 3 Gillberg Neuropsychiatry Centre, University of Gothenburg, Gothenburg, Sweden; 4 Department of Biological Psychology, Vrije Universiteit Amsterdam, Amsterdam, the Netherlands; 5 Child Health Research Centre, University of Queensland, Brisbane, Australia; 6 Child and Youth Mental Health Service, Children’s Health Queensland Hospital and Health Service, Brisbane, Australia; 7 Amsterdam Public Health Research Institute, Amsterdam, the Netherlands

**Keywords:** Adolescence, Young adulthood, Neurodevelopmental disorders, Anxiety disorders, Depressive disorders, Suicidal ideation

## Abstract

**Background:**

Anxiety disorders in adolescence have been associated with several psychiatric outcomes. We sought to describe the prospective relationship between various levels of adolescent anxiety and psychiatric diagnoses (anxiety-, bipolar/psychotic-, depressive-, and alcohol and drug misuse disorders) and suicidal ideation in early adulthood while adjusting for childhood attention-deficit/hyperactivity disorder (ADHD), autism spectrum disorder (ASD), and developmental coordination disorder (DCD). Furthermore, we aimed to estimate the proportion attributable to the various anxiety levels for the outcomes.

**Methods:**

We used a nation-wide population-based Swedish twin study comprising 14,106 fifteen-year-old twins born in Sweden between 1994 and 2002 and a replication sample consisting of 9211 Dutch twins, born between 1985 and 1999. Adolescent anxiety was measured with parental and self-report. Psychiatric diagnoses and suicidal ideation were retrieved from the Swedish National Patient Register and via self-report.

**Results:**

Adolescent anxiety, of various levels, predicted, in the Swedish National Patient Register, anxiety disorders: hazard ratio (HR) = 4.92 (CI 3.33–7.28); depressive disorders: HR = 4.79 (3.23–7.08), and any psychiatric outcome: HR = 3.40 (2.58–4.48), when adjusting for ADHD, ASD, and DCD. The results were replicated in the Dutch data. The proportion of psychiatric outcome attributable to adolescent anxiety over time (age 15–21) was 29% for any psychiatric outcome, 43–40% for anxiety disorders, and 39–38% for depressive disorders.

**Conclusion:**

Anxiety in adolescence constitutes an important risk factor in the development of psychiatric outcomes, revealing unique predictions for the different levels of anxiety, and beyond the risk conferred by childhood ADHD, ASD, and DCD. Developmental trajectories leading into psychiatric outcomes should further empirically investigated.

## Background

Anxiety disorders are an umbrella term for anxiety constructs, for example, social anxiety disorder and panic disorder; and are the most prevalent psychiatric disorders in the western world, with an estimated lifetime prevalence up to 32% [1]. Anxiety disorders often have their onset in childhood or adolescence and frequently co-occur with each other and/or with later psychiatric outcomes such as depression, substance use disorders, and suicidal behavior [2]. In addition, childhood and adolescence anxiety disorders are associated with several adverse functional outcomes in adulthood, such as reduced life satisfaction, familial and social impairment, educational underachievement and poor adjustment at work [2, 3].

Existing studies aimed at investigating the relationship of childhood or adolescent anxiety disorders and adverse adult outcomes have shown robust associations with later risks of adult anxiety-, affective-, substance use-, and behavioral disorders, suicidal behavior, and psychotic symptoms [2–6]. These heterogeneous associations are in line with the emerging concept of a common underlying susceptibility for nearly all mental disorders [7, 8]. In addition, while standardized assessments of psychiatric disorders based on systems such as the Diagnostic and Statistical Manual of Mental Disorders [9] or the International Classification of Diseases [10] are commonly used, accumulating evidence suggests that virtually all psychiatric disorders can best be conceptualized as the extreme end of a dimensionally distributed trait [11], and it is yet unclear how various levels of anxiety are associated with psychiatric outcomes.

Crucially, most longitudinal studies focus on the specific effects of childhood or adolescent anxiety disorders on subsequent psychiatric outcomes without considering that youth anxiety may be confounded by neurodevelopmental disorders such as attention-deficit/hyperactivity disorder (ADHD), autism spectrum disorder (ASD), and developmental coordination disorder (DCD) earlier in childhood. Co-existence between youth anxiety and ADHD [12, 13], ASD [14], and DCD [15] are highly common. Associations between these constructs and psychiatric outcomes, e.g. ADHD and alcohol/substance use disorders [16], or ASD and suicide [17], are considerably stronger than those between anxiety disorders and psychiatric outcomes. Additionally, there is a strong relationship between ADHD and psychiatric disorders that remains after adjustment for anxiety disorders [18]. Finally, neurological soft signs, together with anxious behavior in childhood, were found to be strongly predictive of persistent psychiatric disorder [15], indicating a need to examine the possible confounding of this relationship.

To conclude, current evidence strongly and robustly suggests that youth anxiety disorders predict a broad range of subsequent psychiatric outcomes. However, the risk conferred by various levels of anxiety, as well as ADHD, ASD, and DCD in childhood is yet unknown. This study sought to describe the relationship between different levels of adolescent anxiety and psychiatric diagnoses in late adolescence/young adulthood: alcohol and drug misuse disorders, anxiety disorders, bipolar/psychotic disorders, depressive disorders, and suicidal ideation in a population-based study of 14,106 Swedish twins, replicated in an independent community-based sample comprising 9211 Dutch twins. The specific aims of the study were first, to estimate the association between various levels of adolescent anxiety and later psychiatric diagnoses and suicidal ideation from service-based registers and to test whether ADHD, ASD, and DCD in childhood confound a possible association. Second, to estimate the total amount of psychiatric outcomes attributable to the various levels of adolescent anxiety.

## Method

### Participants

We used two sources to examine the relationship between anxiety in adolescence and psychiatric outcomes in late adolescence/young adulthood: the Child and Adolescent Twin Study in Sweden (CATSS) and the Swedish National Patient Register (NPR). In addition, we conducted replication analyses on a longitudinal dataset that was retrieved from the Netherlands Twin Register (NTR).

### Child and Adolescent Twin Study in Sweden

CATSS is an ongoing, longitudinal, population-based study on somatic and mental health problems in twins during childhood and adolescence. Details can be found elsewhere [19]. Briefly, since 2004, parents of all Swedish twins born from July 1992 and onwards are contacted in connection with the twins’ ninth or 12th birthday (CATSS-9/12) and asked to participate in a telephone interview, which includes measures of, among other things, neurodevelopmental disorders. When the twins reach the age of 15 (CATSS-15), families are contacted again and asked to fill out a web-based questionnaire, targeting various mental health problems and social milieus. CATSS-15 includes twins born from the first of January 1994 up until 2002. In the present study, we included 12,324 twins who completed the self-reports at age 15, and parental report covering 11,133 twins, out of which a total of 85% (self-report) and 87% (parental report) had complete data at age 9/12. A total of 14,106 twins where covered with either self- or parental report.

### Measures

#### Anxiety in adolescence

CATSS-15 includes both the parental and self-report version of the Strengths and Difficulties Questionnaire (SDQ; [20]). The SDQ is a brief behavioral screening questionnaire for children and adolescents between the ages of 3 to 16 years. In the present study, the emotional problems scale, parental and self-report, was used as an indicator of anxiety in adolescence. The scale has 5 items with 3 response options: 0 (not true), 1 (somewhat true), and 2 (certainly true). In addition to dimensional scoring, three categories [20] have been proposed so that roughly 80% of the children fall into a ‘normal’ (in the present study scores between 0 and 2 in the parental report, scores between 0 and 4 in the self-report), 10% into a ‘borderline’ (i.e. score 3 in the parental report, score 5 in the self-report) and 10% into an ‘abnormal’ (i.e. ≥ 4 in the parental report, ≥ 6 in the self-report) category. The emotional problems scale has been reported to have a sensitivity/specificity ranging between 29/96% (youth report) to 54/91% (parent report) for anxiety disorders [21] and correlates strongly with other measures of anxiety in youth [22]. In the present study, the Area Under the Receiving Characteristics Curve for the scale was 0.67/0.69 when comparing parental/self-report to the clinical diagnosis of anxiety disorder from the register mentioned below. Studies of the Swedish version of the SDQ symptom scales have confirmed the factor structure of the original English SDQ [23], and results from a validation study of the Swedish SDQ indicate that the parental version has a good discriminatory validity [24].

#### Neurodevelopmental disorders

The Autism-Tics, ADHD, and other Comorbidities inventory (A-TAC [25]; is included in CATSS-9/12. The A-TAC is a fully structured 96-item parent-report telephone interview designed for large-scale epidemiological purposes. To screen for neurodevelopmental disorders in childhood, the A-TAC is based on symptom criteria and common clinical features. Items are scored as 1 (yes), 0.5 (yes, to some extent), and 0 (no) and are divided into modules corresponding to diagnostic domains. Cross-sectional and longitudinal validation studies show good to excellent predictive validity for, amongst others, ADHD, ASD, and DCD [26, 27]. In this study, cut-offs ≥6 for ADHD, ≥ 4.5 for ASD, and ≥ 0.5 for DCD with a sensitivity of (0.91/0.91/0.63) and a specificity of (0.73/0.80/0.68) were used as a screening cut-off for ADHD, ASD, and DCD.

#### Psychiatric outcomes in late adolescence/young adulthood

A personal identification number, which is assigned to all individuals living in Sweden either at birth or when permanently moving to Sweden, enables linkage across health and service registers. The Swedish National Patient Register (NPR) includes all inpatient data from 1987, and outpatient data from 2001 and is coded according to ICD-9 and 10 codes. The validity of the NPR is continuously assessed. Several studies report high validity and reliability of several disorders, e.g., bipolar disorder [28] and psychotic disorders [29]. The following ICD-10 codes were retrieved for all participants and are commonly referred to as ‘psychiatric outcomes’ in this study: F10–19 (alcohol and drug misuse disorders), F20–29 and F30–31 (psychotic disorders and bipolar disorders were merged into one category), F32–39 (depressive disorders), F40–41 (anxiety disorders), and X60–84 (suicidal ideation and suicide attempts) including all subgroups. In our linkage, the NPR was updated until the 31st of December 2014. For each individual, we recorded ICD-10 codes and age at first observed event. Any individual could have multiple outcomes but was still analyzed separately for each outcome. The overlap can be found in Additional file 1: Table S1. Further, we calculated a sum of outcomes, yielding values between 0 and 5.

### Sensitivity analysis and attrition

To assess whether missing values affected our estimates and inferences, we used multiple imputation with chained equations to handle missing values in variables, as implemented in the R-package ‘mice’ [30]. Values were imputed using a random forest approach. We analyzed the associations between self- and parental reported anxiety and the 5 separate outcomes, as well as the collapsed, for the Swedish sample. No significant changes were found (Additional file 1: Table S2). Prevalence of psychiatric outcomes in individuals whose parents responded only at age 9/12 but not at age 15, versus those who responded at both assessment waves are characterized in Additional file 1: Table S3. The prevalence differed significantly in responders and non-responders (4.2 and 8.5%, χ^2^ = 153.56, *p* < 0.001).

### Statistical analyses

We excluded individuals with psychiatric outcomes assigned before the age of 15 (*N* = 181) from all analyses and used log-linear regression models with the total number of psychiatric diagnoses (alcohol and drug misuse disorders, anxiety disorders, bipolar/psychotic disorders, depressive disorders) and suicidal ideation as the dependent variable, and the total score of anxiety as the independent variable (ranging from 0 to 10). Results are presented as rate ratios (RR), i.e. the increase in rate of the outcome, per unit increase in the exposure. Next, we used Cox proportional hazard regression to regress each of the specific psychiatric outcomes on anxiety (unadjusted model) and excluded individuals who were assigned the respective psychiatric outcome before the age of 15. Results are presented as hazard ratios, i.e. comparing the risk of the outcome in the exposed and unexposed group while accounting for follow-up time. Parental report and self-report in the independent variable were analyzed separately. We entered ADHD, ASD, and DCD cut-offs, as well as sex into the models as covariates in order to test for confounding.

A cluster-robust sandwich estimator was applied to adjust the standard errors for the nested twin data. For our collapsed outcome “any psychiatric outcome”, *p* < 0.05 was considered statistically significant. For the individual psychiatric outcomes, i.e., alcohol and drug misuse disorders, anxiety disorders, bipolar/psychotic disorders, depressive disorders, and suicidal ideation (unadjusted and adjusted for ADHD, ASD, DCD, and sex, yielding 10 tests), we set the statistical significance threshold at *p* < 0.005 to account for multiple comparisons. Estimates for anxiety categories ‘borderline’ and ‘abnormal’ were calculated relative to the anxiety category ‘normal’, which served as the reference category.

To account for unmeasured confounders, (a) we conducted a within-twin analysis, which capitalizes on the complete genetic relatedness of monozygotic twin pairs. The design takes genetic and environmental confounding into account by comparing the risk of the outcome in twins differentially exposed (i.e., having different levels of anxiety [31]), (b) we calculated the E-value, which is the lowest point estimate of both the confounder associations and the measured covariates that needs to be present in order for an (unmeasured) confounder to fully explain the association [32].

#### Survival analyses and attributable fractions

We created survival curves based on the unadjusted model using the R function ‘survfit’ from the package ‘survival’ [33]. We then estimated the total amount, while adjusting for childhood ADHD, ASD, and DCD, as well as sex, of psychiatric outcomes in late adolescence/young adulthood that could be attributed to anxiety by calculating the attributable fraction (AF) using the ‘AFcoxph’ function from the package ‘AF’ [34] in R. The ‘AFcoxph’ function allows the AF to vary over time and thus renders it possible to estimate age-specific AFs. The function estimates the model-based adjusted attributable fraction from the Cox proportional hazard regression model and is commonly used for data from cohort sampling designs with time-to-event outcomes while adjusting for potential confounders. All analyses were performed in R version 3.5.1 [35].

### Replication from the Netherlands Twin Register

The Young Netherlands Twin Register (YNTR) and the Adult Netherlands Twin Register (ANTR) have been described in detail elsewhere [36, 37]. The sample included twins who were born the 1st January 1985 to the 31st December 1999 and were part of the YNTR. Nine thousand two hundred eleven twins were assessed at age 14 and then followed up at age 16 and 18. The retention rate varied between 10 and 47%, depending on follow up, giving sample sizes between 914 and 4283. In order to mirror the analyses from CATSS, we used the empirically based anxious/depressed syndrome scale scored from the Youth Self-Report [38] to create the exposure measure. The distribution of anxiety levels was carried out in the same fashion as in the main analyses (80% ‘normal’, 10% ‘borderline’, 10% ‘abnormal’). As outcomes, we used the Achenbach DSM-oriented scales at age 16: anxiety (“anxiety problems”, 6 items), and depression (“affective problems”, 13 items) and 18: for bipolar/psychotic disorders, we used the Adult Self-Report (ASR) Thought Problems scale [39]. For alcohol and drug misuse disorders, a total score of 16 or higher on the World Health Organization’s Alcohol Use Disorders Identification Test [40], implying high-risk/harmful alcohol use, was used. For suicidal ideation as an outcome, we used an individual question from the survey in the ASR indicating suicidal ideation: “I deliberately try to hurt or kill myself”, answered with either “somewhat or sometimes” or “very much so or often”. A dichotomization was conducted at the 90th percentile for all scales to classify individuals as screen-positive for the respective outcome. As covariates, the ten items ASD scales at age 7, 10 and 12, developed by So et al. [41], and the ten items Attention Problems scales at age 7, 10, and 12, developed by Achenbach and Rescorla [38], both from the Child Behavior Checklist, were included. As the outcome variables in the NTR consisted of self-report and we had no access to follow-up time, we used logistic regression models to estimate the association between exposure and outcome and included ADHD and ASD cut-offs, as well as sex, as covariates into the model. Results are presented as odds ratios. A cluster-robust sandwich estimator was applied to adjust for standard errors for the nested twin data. *P* < .05 was considered statistically significant. For the individual psychiatric outcomes, i.e., high-risk/harmful alcohol use, anxiety, thought problems, depression, and suicidal ideation (unadjusted and adjusted for ADHD, ASD, and sex, yielding 10 tests), we set the statistical significance threshold at *p* < 0.005 to account for multiple comparisons. Estimates for anxiety categories ‘borderline’ and ‘abnormal’ were calculated relative to the anxiety category ‘normal’, which served as the reference category.

## Results

### Descriptives

Table 1 shows the prevalence and distribution of psychiatric diagnoses and suicidal ideation in self-report and parental report, among the three anxiety categories in CATSS-15. One in every thirty-three twins who filled out the SDQ received a registry-based psychiatric outcome during the follow-up period. Anxiety disorders were the most prevalent psychiatric outcome (1.4%), followed by depressive disorders (1.3%), and alcohol and drug misuse disorders (1.0%). Both for parental and self-reports, individuals in the ‘borderline’ and ‘abnormal’ anxiety categories were overall more commonly assigned a psychiatric outcome during the follow-up period, compared to individuals in the ‘normal’ anxiety category. Sex-specific prevalences and distribution of psychiatric outcomes for the whole sample can be found in Additional file 1: Table S4 and showed that females were more commonly diagnosed with any psychiatric outcome, anxiety disorders, and depressive disorders.

**Table 1:** Prevalence and distribution of psychiatric outcomes at follow up among ‘normal’, ‘borderline’ and ‘abnormal’ anxiety categories at age 15 self-report and parental report

	Anxiety category			
	Total N (%)	Normal	Borderline	Abnormal
Self-report	12,324 (100)	9359 (75.9)	1099 (8.9)	1866 (15.1)
No psychiatric outcome	11,950 (97.0)	9139 (97.6)	1056 (96.1)	1755 (94.1)
Any psychiatric outcome	374 (3.0)	220 (2.4)	43 (3.9)	111 (5.9)
Alcohol and drug misuse disorders	126 (1.0)	93 (1.0)	14 (1.3)	19 (1.0)
Anxiety disorders	175 (1.4)	83 (0.9)	28 (2.5)	64 (3.4)
Bipolar/psychotic disorders	16 (0.1)	12 (0.1)	2 (0.2)	2 (0.1)
Depressive disorders	157 (1.3)	76 (0.8)	16 (1.5)	65 (3.5)
Suicidal ideation	40 (0.3)	23 (0.2)	4 (0.4)	13 (0.7)
Parental report	11,133 (100)	9230 (82.9)	829 (7.4)	1074 (9.6)
No psychiatric outcome	10,775 (96.8)	9023 (97.8)	782 (94.3)	970 (90.3)
Any psychiatric outcome	358 (3.2)	207 (2.2)	47 (5.7)	104 (9.7)
Alcohol and drug misuse disorders	117 (1.1)	82 (0.9)	11 (1.3)	24 (2.2)
Anxiety disorders	167 (1.5)	82 (0.9)	25 (3.0)	60 (5.6)
Bipolar/psychotic disorders	16 (0.1)	12 (0.1)	2 (0.2)	2 (0.2)
Depressive disorders	146 (1.3)	74 (0.8)	25 (3.0)	47 (4.4)
Suicidal ideation	36 (0.3)	22 (0.2)	3 (0.4)	11 (1.0)

### Regression analyses

Each one-point increase on the total score of anxiety at age 15, based on self-report, corresponded to a 23% elevation in the number of assigned psychiatric outcomes (self-report: RR = 1.23, 95% CI 1.17–1.29; parental report: RR = 1.37, 95% CI 1.31–1.42). This held also true after adjusting for sex, ADHD, ASD, DCD in childhood (self-report: RR = 1.20, 95% CI 1.14–1.27; parental report: RR = 1.32, 95% CI 1.25–1.39).

Table 2 shows the associations between adolescent anxiety categories and the various psychiatric outcomes for parental and self-report retrieved from the Cox proportional hazard ratio analyses. Adjusted HRs for the outcome of any psychiatric diagnosis or suicidal ideation were 1.73 (95% CI 1.20–2.49; self-report) and 2.32 (95% CI 1.64–3.29; parental report) in the ‘borderline’ categories and 3.40 (95% CI 2.58–4.48; self-report) and 3.88 (95% CI 2.86–5.25; parental report) in the ‘abnormal’ anxiety categories. The ‘borderline’ and ‘abnormal’ anxiety categories across both sources of report were significantly associated with anxiety disorders and depressive disorders, with the exception of the ‘borderline’ categories for depressive disorders (both unadjusted and adjusted, self-report). Within-twin analyses conducted on monozygotic twins primarily yielded non-significant effects, with very wide CI:s, for the association between adolescent anxiety categories and psychiatric outcomes (see Additional file 1: Table S5). Corresponding E-values can be found in Additional file 1: Table S6 and showed that, overall, the E-values were roughly twice as high as all the significant hazard ratios, indicating that substantial confounding must be present in order to completely explain away the observed effect.

**Table 2:** Associations between anxiety category at baseline and psychiatric outcome in self-report and parental report. Figures are hazard ratios (95% confidence intervals)

	Anxiety category					
	Unadjusted			Adjusted^a^		
Psychiatric outcome	Normal^b^	Borderline	Abnormal	Normal^b^	Borderline	Abnormal
Self-report						
Any psychiatric outcome	1	**1.90 (1.36–2.65)**	**3.72 (2.93–4.72)**	1	**1.73 (1.20–2.49)**	**3.40 (2.58–4.48)**
Alcohol and drug misuse disorders	1	1.44 (0.82–2.54)	1.42 (0.87–2.32)	1	1.22 (0.62–2.38)	1.25 (0.69–2.25)
Anxiety disorders	1	**3.35 (2.18–5.16)**	**5.74 (4.15–7.96)**	1	**2.67 (1.65–4.34)**	**4.92 (3.33–7.28)**
Bipolar/psychotic disorders	1	1.61 (0.38–7.26)	1.18 (0.26–5.30)	1	1.90 (0.46–7.87)	1.54 (0.39–6.04)
Depressive disorders	1	2.03 (1.18–3.50)	**6.14 (4.39–8.58)**	1	1.58 (0.87–2.88)	**4.79 (3.23–7.08)**
Suicidal ideation	1	1.68 (0.58–4.85)	**3.93 (2.05–7.55)**	1	1.16 (0.35–3.84)	2.33 (1.04–5.22)
Parental report						
Any psychiatric outcome	1	**2.60 (1.88–3.59)**	**4.88 (3.78–6.29)**	1	**2.32 (1.64–3.29)**	**3.88 (2.86–5.25)**
Alcohol and drug misuse disorders	1	1.55 (0.82–2.91)	**2.85 (1.80–4.49)**	1	1.54 (0.79–3.02)	2.15 (1.21–3.83)
Anxiety disorders	1	**3.60 (2.30–5.64)**	**7.52 (5.36–10.56)**	1	**3.11 (1.92–5.04)**	**6.01 (4.05–8.93)**
Bipolar/psychotic disorders	1	1.95 (0.43–8.71)	1.59 (0.35–7.17)	1	1.72 (0.38–7.75)	1.08 (0.30–3.87)
Depressive disorders	1	**4.02 (2.57–6.29)**	**6.52 (4.43–9.61)**	1	**3.32 (2.05–5.37)**	**5.07 (3.20–8.03)**
Suicidal ideation	1	1.57 (0.47–5.26)	**4.88 (2.37–10.07)**	1	1.61 (0.46–5.63)	**3.76 (1.62–8.72)**

^a^ Adjusted for sex, attention-deficit/hyperactivity disorder, autism spectrum disorder, and developmental coordination disorder at age 9/12. Bold estimates indicate significance at the 0.005 level, except for ‘any psychiatric outcome’, where alpha was set to 0.05.

^b^ Reference category

### Survival analyses and attributable fractions

**Fig. 1:**
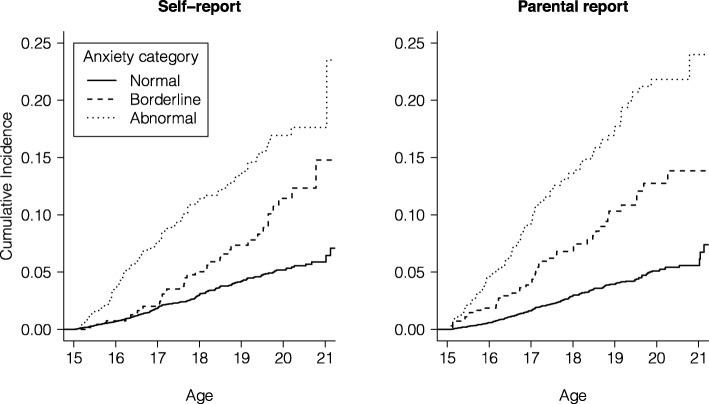
Cumulative incidence of any psychiatric outcome in individuals within ‘normal’, ‘borderline’, and ‘abnormal’ anxiety categories at age 15 in self-report and parental report. *Note.* For representational purposes, figures were cut at age 21

Survival curves were converted depicting cumulative incidence conditioned on level of anxiety. Figure 1 shows age-dependent cumulative incidence of any psychiatric outcome in self-report and parental report in individuals within the three anxiety categories. The overall cumulative incidence for any outcome up to the end of follow-up (age 21) for those who self-reported were 7, 15 and 25% in the ‘normal, ‘borderline’, and ‘abnormal’ group. A similar pattern could be seen for parental reports. For anxiety disorders specifically, the overall cumulative incidence up to the end of follow-up was 5% in individuals with ‘normal’ anxiety, 9% in individuals with ‘borderline’ anxiety and 17% in individuals with ‘abnormal’ anxiety in individuals who self-reported. Estimates for all specific psychiatric outcomes can be found in the additional material (Additional files 2, 3, 4, 5, 6: Figures S1-S5). The incidence of any psychiatric outcome increased steadily for the ‘normal’ anxiety category, whereas ‘borderline’ and ‘abnormal’ anxiety categories increased steeply, with a possible inflection point occurring around 19 years of age.

**Fig. 2:**
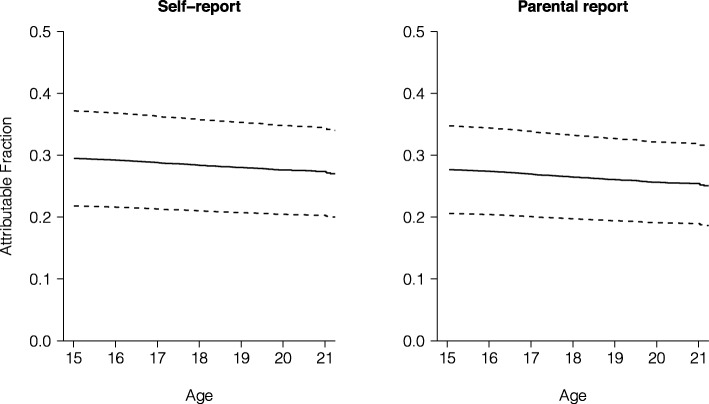
Attributable fraction of any psychiatric outcome in individuals within ‘borderline’ and ‘abnormal’ anxiety categories at age 15 in self−report and parental report. *Note.* Dashed lines represent 95% confidence intervals. For representational purposes, figures were cut at age 21

Figure 2 displays the attributable fractions (AFs) for any psychiatric outcome until late adolescence/young adulthood that is attributable to ‘borderline’ and ‘abnormal’ adolescence anxiety at age 15 in self-report and parental report, suggesting that, over time, 29% of the occurrence of any psychiatric outcome is attributable to prior adolescent anxiety (self-report). Consistent with the magnitude of the HRs for specific outcomes, the highest AFs were found for anxiety disorders (AF 43–40%), depressive disorders (AF 39–38%), and suicidal ideation (AF 21%, all self-report; see additional material for (Additional files 7, 8, 9, 10, 11: Figures S6-S10 on all specific outcomes).

### Replication in the NTR

Prevalence and distributions from the NTR are reported in Additional file 1: Table S7 in the additional material. High prevalences were found for thought problems, anxiety, and depression. The results from the log-linear regression analysis showed a similar pattern as in CATSS, both in the unadjusted model (RR = 1.18, 95% CI 1.17–1.19), and when adjusted for sex, ADHD and ASD in childhood (RR = 1.16, 95% CI 1.15–1.17). Results from the logistic regression models were similar to what was seen in the CATSS albeit with stronger associations, i.e. the highest risks were found in the ‘abnormal’, followed by the ‘borderline’ category, for anxiety (OR = 14.02, 95% CI 10.38–18.93), and depression (OR = 8.00, 95% CI 5.93–10.79; see Additional file 1: Table S8 for all specific outcomes).

## Discussion

In this population-based longitudinal study, adolescent anxiety, of various levels, was found to elevate the risk of developing a range of subsequent psychiatric outcomes in late adolescence/young adulthood. Highest risk elevations were found for anxiety disorders (7.52), depressive disorders (6.52), any psychiatric outcome (4.88), and suicidal ideation (4.88). All associations remained virtually unaltered after adjusting for ADHD, ASD, and DCD. Risk patterns between anxiety in adolescence and later psychiatric outcomes were mirrored in the Dutch sample, regardless of differences in reports and instruments.

Incidence patterns revealed distinct properties of the three anxiety categories that affected the risk of developing psychiatric outcomes. Namely, there was steep, increased risk for the ‘borderline’ and ‘abnormal’ anxiety categories. Cumulative incidence was highest in the ‘abnormal’ anxiety category for any psychiatric outcome (25%), anxiety disorders (17%), alcohol and drug misuse disorders (12%), and depressive disorders (10%). For both individuals in the ‘borderline’ and ‘abnormal’ anxiety group, the proportion of psychiatric outcome that could theoretically be avoided if adolescent anxiety was prevented or treated early was highest for anxiety disorders and depressive disorders (~ 40%). AFs further indicate that adolescence anxiety accounted for up to a third of any assessed psychiatric diagnosis in late adolescence/young adulthood.

Our finding that adolescent anxiety predicts young adult psychiatric outcomes beyond the risk conferred by childhood ADHD, ASD and DCD was not anticipated. These constructs are generally stronger predictors for psychiatric outcomes than anxiety [16, 17] and the onset almost always precedes that of anxiety disorders. In a recent study, Rice et al. [42] delineated two age of onset based trajectories to Major Depressive Disorder. The early-adolescence onset group was associated with a genetic risk for neurodevelopmental disorders, while the later-adolescence-onset group was associated with genetic risk for Major Depressive Disorder only. We therefore conducted additional post-hoc analyses including the 181 individuals who received their diagnosis before the age of 15. No meaningful difference could be discerned (HR for any outcome, ‘abnormal’ anxiety category, self-report: 3.45; parental report: 4.77) suggesting that our results were not confounded by the exclusion of a possible early-adolescence onset group characterized by neurodevelopmental disorder susceptibility.

The ‘borderline’ anxiety categories predicted a broad range of our assessed outcomes in late adolescence/young adulthood. That is, at group level, adolescents in the ‘borderline’ anxiety category both had an earlier onset and higher rates of early adult psychiatric outcomes. This suggests that a screening based on symptom presentation of anxiety rather than on full diagnostic criteria should always be conducted in clinical settings, irrespective of the focus of the assessment. Clinicians should remain vigilant towards those reporting elevated levels of anxiety, as incidence patterns for the specific psychiatric outcomes for the ‘borderline’ anxiety group were all characterized by steep curves.

The incidence of psychiatric outcomes were roughly evenly distributed over time, suggesting that there is no clearly identifiable time point when an individual with elevated levels of anxiety is susceptible to be diagnosed with a psychiatric disorder. In self-report data from ages 15 to 17, the ‘borderline’ anxiety group could not be distinguished from the ‘normal’ anxiety group, so parental report might therefore be preferable in clinical settings for this age group in order to identify individuals with an elevated risk for psychiatric outcomes based on their levels of anxiety. While a more detailed delineation of trajectories of individuals with anxiety is undoubtedly necessary, five questions administered at age 15 were sufficient to prospectively identify a third of all assessed psychiatric outcomes. Studies on the efficacy of treatment for anxiety disorders have reported an observed reduction in associated symptomatology and comorbidity, including depression, and other anxiety disorders [43, 44]. Thus, it is possible that treatments directed towards the core symptoms of anxiety in adolescence may attenuate the risk and affect the trajectories leading into a wide array of psychiatric outcomes (i.e. anxiety disorders, depressive disorders, alcohol and drug misuse disorders, possibly also bipolar/psychotic disorders) and suicidal ideation. As a final note, even though the within-twin design was underpowered and accompanied by very wide CI:s, the results may indicate that the association between anxiety and psychiatric outcomes can be confounded by shared environmental and/or genetic factors. This highlights the importance of studying the development of anxiety using genetically sensitive designs.

The study has several strengths which include large sample sizes, high response rates, the use of a validated assessment instrument, the usage of two independent population-based samples, and finally, linkage to a nation-wide register, which offers the possibility to analyze anxiety and psychiatric outcomes with little or no shared method variance, while also circumventing the problem of attrition.

The results also have to be interpreted in the light of limitations. Participants were followed up until either the occurrence of the first psychiatric outcome or when the coverage of the NPR entries ended, around the age of 22 for the oldest participants. Thus, the rate of false negatives might be elevated. As a consequence, the estimates for disorders which generally have a later mean age of onset, e.g., bipolar and psychotic disorders, should therefore be interpreted with caution. Related, the NPR only includes diagnoses in out or in-patient care, why diagnoses assigned in primary care, reasonably those with less profound symptomatology, are not included in the analyses. Difference in response rate from ages 9 and 15 may have affected the observed association, as reflected in the attrition analyses. Thus, the generalizability should be interpreted with caution. We can only speculate about the direction of the effect but it seems plausible that, if anything, it attenuated the observed associations. Finally, some exposure misclassification should be expected as the SDQ does not have perfect sensitivity and specificity nor does the anxiety category ‘abnormal’ equate a clinical construct as, for instance, the duration of the distress and impairment arising from the symptoms are not assessed or accounted for. This is evident as prevalence estimates from epidemiological studies generally are higher than clinical estimates [45].

## Conclusion

In conclusion, the results of the present study suggest that anxiety in adolescence constitutes an important risk factor in the development of psychiatric diagnoses and suicidal ideation, and revealed unique predictions for the different levels of anxiety, and beyond the risk conferred by childhood ADHD, ASD and DCD. This emphasizes the need to further empirically investigate developmental trajectories leading from neurodevelopmental disorders and anxiety into adverse outcomes later in life.

## Supplementary information

**Additional file 1: Table S1.** Overlap of psychiatric outcomes for individuals in the Swedish National Patient Register (NPR). **Table S2**. Associations between anxiety category at baseline and psychiatric outcome in self-report and parental report after multiple imputation for variables ADHD, ASD, DCD, and anxiety self- and parental report. **Table S3** Prevalence and distribution of psychiatric outcomes in individuals whose parents only responded at CATSS-9/12 but not at CATSS-15, versus those who have responded at both assessment waves. **Table S4** Prevalence and distribution of psychiatric outcomes divided by sex. **Table S5** Associations between anxiety category at baseline and psychiatric outcome in CATSS, estimated from within-twin analyses conducted on monozygotic twins. **Table S6** E-values for the associations between anxiety category at baseline and psychiatric outcome in self-report and parental report. **Table S7**. Prevalence and distribution of psychiatric outcomes in the Netherlands Twin Register (NTR) among anxiety categories ‘normal’, ‘borderline’, and ‘abnormal’. **Table S8**. Associations between anxiety category at baseline and psychiatric outcome in the NTR.

**Additional file 2: Figure S1.** Cumulative incidence of alcohol and drug misuse disorders in individuals within 'normal', 'borderline', and 'abnormal' anxiety categories at age 15 in self-report and parental report. *Note.* For representational purposes, figures were cut at age 21.

**Additional file 3: Figure S2.** Cumulative incidence of anxiety disorders in individuals within 'normal', 'borderline', and 'abnormal' anxiety categories at age 15 in self-report and parental report. *Note.* For representational purposes, figures were cut at age 21.

**Additional file 4: Figure S3.** Cumulative incidence of bipolar/psychotic disorders in individuals within 'normal', 'borderline', and 'abnormal' anxiety categories at age 15 in self-report and parental report. *Note.* For representational purposes, figures were cut at age 21.

**Additional file 5: Figure S4.** Cumulative incidence of depressive disorders in individuals within 'normal', 'borderline', and 'abnormal' anxiety categories at age 15 in self-report and parental report. *Note.* For representational purposes, figures were cut at age 21.

**Additional file 6: Figure S5.** Cumulative incidence of suicidal ideation in individuals within 'normal', 'borderline', and 'abnormal' anxiety categories at age 15 in self-report and parental report. *Note.* For representational purposes, figures were cut at age 21.

**Additional file 7: Figure S6.** Attributable fraction of alcohol and drug misuse disorders in individuals within 'borderline' and 'abnormal' anxiety categories at age 15 in self-report and parental report. *Note.* Dashed lines represent 95% confidence intervals. For representational purposes, figures were cut at age 21.

**Additional file 8: Figure S7.** Attributable fraction of anxiety disorders in individuals within 'borderline' and 'abnormal' anxiety categories at age 15 in self−report and parental report. *Note.* Dashed lines represent 95% confidence intervals. For representational purposes, figures were cut at age 21.

**Additional file 9: Figure S8.** Attributable fraction of bipolar/psychotic disorders in individuals within 'borderline' and 'abnormal' anxiety categories at age 15 in self−report and parental report. *Note.* Dashed lines represent 95% confidence intervals. For representational purposes, figures were cut at age 20.

**Additional file 10: Figure S9.** Attributable fraction of depressive disorders in individuals within 'borderline' and 'abnormal' anxiety categories at age 15 in self−report and parental report. *Note.* Dashed lines represent 95% confidence intervals. For representational purposes, figures were cut at age 21.

**Additional file 11: Figure S10.** Attributable fraction of suicidal ideation in individuals within 'borderline' and 'abnormal' anxiety categories at age 15 in self−report and parental report. *Note.* Dashed lines represent 95% confidence intervals. For representational purposes, figures were cut at age 21.

## Data Availability

The datasets are available on reasonable request from the corresponding author at sabrina.doering@gu.se.

## Abbreviations

ADHD: Attention-deficit/hyperactivity disorder
ANTR: Adult Netherlands Twin Register
ASD: Autism spectrum disorder
ASR: Adult Self-Report
A-TAC: Autism-Tics, ADHD, and other Comorbidities inventory
CATSS: Child and Adolescent Twin Study in Sweden
DCD: Developmental coordination disorder
NPR: Swedish National Patient Register
NTR: Netherlands Twin Register
SDQ: Strengths and Difficulties Questionnaire
