# Supplementary material for: Anxiety at age 15 predicts psychiatric diagnoses and suicidal ideation in late adolescence and young adulthood: results from two longitudinal studies
Source: BMC Psychiatry. 2019 Nov 14;19:363. doi: 10.1186/s12888-019-2349-3 (PMC6857289; doi:10.1186/s12888-019-2349-3)
Supplement: Supplementary file 1 — Additional file 1: Table S1. Overlap of psychiatric outcomes for individuals in the Swedish National Patient Register (NPR). Table S2. Associations between anxiety category at baseline and psychiatric outcome in self-report and parental report after multiple imputation for variables ADHD, ASD, DCD, and anxiety self- and parental report. Table S3 Prevalence and distribution of psychiatric outcomes in individuals whose parents only responded at CATSS-9/12 but not at CATSS-15, versus those who have responded at both assessment waves. Table S4 Prevalence and distribution of psychiatric outcomes divided by sex. Table S5 Associations between anxiety category at baseline and psychiatric outcome in CATSS, estimated from within-twin analyses conducted on monozygotic twins. Table S6 E-values for the associations between anxiety category at baseline and psychiatric outcome in self-report and parental report. Table S7. Prevalence and distribution of psychiatric outcomes in the Netherlands Twin Register (NTR) among anxiety categories ‘normal’, ‘borderline’, and ‘abnormal’. Table S8. Associations between anxiety category at baseline and psychiatric outcome in the NTR. [file 12888_2019_2349_MOESM1_ESM.docx]

**Additional material**

1. Table S1: Overlap of psychiatric outcomes for individuals in the Swedish National Patient Register (NPR).
2. Table S2: Associations between anxiety category at baseline and psychiatric outcome in self-report and parental report after multiple imputation for variables ADHD, ASD, DCD, and anxiety self- and parental report.
3. Table S3: Prevalence and distribution of psychiatric outcomes in individuals whose parents only responded at CATSS-9/12 but not at CATSS-15, versus those who have responded at both assessment waves.
4. Table S4: Prevalence and distribution of psychiatric outcomes divided by sex.
5. Table S5: Associations between anxiety category at baseline and psychiatric outcome in CATSS, estimated from within-twin analyses conducted on monozygotic twins.
6. Table S6: E-values for the associations between anxiety category at baseline and psychiatric outcome in self-report and parental report.
7. Table S7: Prevalence and distribution of psychiatric outcomes in the Netherlands Twin Register (NTR) among anxiety categories ‘normal’, ‘borderline’, and ‘abnormal’.
8. Table S8: Associations between anxiety category at baseline and psychiatric outcome in the NTR.
9. Figure S1 – S5: Age-dependent cumulative incidence of alcohol and drug misuse disorders, anxiety disorders, bipolar/psychotic disorders, depressive disorders, and suicidal ideation, respectively.
10. Figure S6 – S10: Standardized attributable risk of alcohol and drug misuse disorders, anxiety disorders, bipolar/psychotic disorders, depressive disorders, and suicidal ideation, respectievely.

| **Table S1.** Overlap of psychiatric outcomes for individuals in the Swedish National Patient Register (NPR). | | | | | |
| --- | --- | --- | --- | --- | --- |
|  | Alcohol and drug misuse disorders | Anxiety disorders | Bipolar/psychotic disorders | Depressive disorders | Suicidal ideation |
| Alcohol and drug misuse disorders | 143 |  |  |  |  |
| Anxiety disorders | 30 | 200 |  |  |  |
| Bipolar/psychotic disorders | 5 | 6 | 20 |  |  |
| Depressive disorders | 21 | 77 | 5 | 177 |  |
| Suicidal ideation | 14 | 23 | * | 22 | 48 |
| *Fewer than five | | | | | |

| **Table S2**. Associations between anxiety category at baseline and psychiatric outcome in self-report and parental report after multiple imputation for variables ADHD, ASD, DCD, and anxiety self- and parental report. Figures are hazard ratios (95% confidence intervals). | | | | | | | |
| --- | --- | --- | --- | --- | --- | --- | --- |
|  | Anxiety category | | | | | | |
|  | Unadjusted | | |  | Adjusted* | | |
| Psychiatric outcome | Normal† | Borderline | Abnormal |  | Normal† | Borderline | Abnormal |
| **Self-report** |  |  |  |  |  |  |  |
| Any psychiatric outcome | 1 | **1.89 (1.35–2.64)** | **3.56 (2.82–4.49)** |  | 1 | **1.78 (1.26–2.50)** | **3.14 (2.46–4.01)** |
| Alcohol and drug misuse disorders | 1 | 1.40 (0.81–2.42) | 1.37 (0.85–2.23) |  | 1 | 1.39 (0.80–2.43) | 1.35 (0.82–2.23) |
| Anxiety disorders | 1 | **3.14 (2.01–4.91)** | **5.48 (3.99–7.52)** |  | 1 | **2.81 (1.79–4.42)** | **4.68 (3.34–6.56)** |
| Bipolar/psychotic disorders | 1 | 1.78 (0.37–8.58) | 1.21 (0.27–5.43) |  | 1 | 2.04 (0.40–10.40) | 1.41 (0.28–7.03) |
| Depressive disorders | 1 | 1.91 (1.11–3.29) | **5.62 (4.08–7.74)** |  | 1 | 1.68 (0.97–2.91) | **4.62 (3.28–6.49)** |
| Suicidal ideation | 1 | 1.60 (0.54–4.75) | **3.51 (1.78–6.94)** |  | 1 | 1.13 (0.38–3.38) | 2.28 (1.12–4.66) |
| **Parental report** |  |  |  |  |  |  |  |
| Any psychiatric outcome | 1 | **2.45 (1.79–3.35)** | **4.36 (3.35–5.67)** |  | 1 | **2.24 (1.63–3.07)** | **3.67 (2.77–4.85)** |
| Alcohol and drug misuse disorders | 1 | 1.37 (0.75–2.51) | **2.57 (1.64–4.02)** |  | 1 | 1.33 (0.73–2.32) | **2.34 (1.47–3.88)** |
| Anxiety disorders | 1 | **3.21 (2.06–5.02)** | **6.55 (4.71–9.12)** |  | 1 | **2.90 (1.85–4.55)** | **5.50 (3.87–7.81)** |
| Bipolar/psychotic disorders | 1 | 1.50 (0.34–6.57) | 1.55 (0.36–6.64) |  | 1 | 1.31 (0.30–5.79) | 0.93 (0.21–4.25) |
| Depressive disorders | 1 | **3.52 (2.29–5.44)** | **5.57 (3.91–7.93)** |  | 1 | **3.12 (2.02–4.82)** | **4.51 (3.10–6.55)** |
| Suicidal ideation | 1 | 1.44 (0.44–4.68) | **3.81 (1.78–8.14)** |  | 1 | 1.17 (0.36–3.88) | 2.50 (1.17–5.38) |
| * Adjusted for sex, attention-deficit/hyperactivity disorder, autism spectrum disorder, and developmental coordination disorder at age 9/12. Bold estimates indicate significance at the 0.005 level, except for ‘any psychiatric outcome’, where alpha was set to 0.05.  † Reference category. | | | | | | | |

| **Table S3**. Prevalence and distribution of psychiatric outcomes in individuals whose parents only responded at age 9/12 (CATSS-9/12) but not at age 15 (CATSS-15), versus those who have responded at both assessment waves. | | | |
| --- | --- | --- | --- |
|  | CATSS-9/12 (%) | CATSS-15 (%) | P-value* |
|  | 6129 (100) | 14106 (100) |  |
| No psychiatric outcome | 5609 (91.5) | 13519 (95.8) | <0.001 |
| Any psychiatric outcome | 520 (8.5) | 587 (4.2) | <0.001 |
| Alcohol and drug misuse disorders | 173 (2.8) | 177 (1.3) | <0.001 |
| Anxiety disorders | 260 (4.2) | 283 (2.0) | <0.001 |
| Bipolar/psychotic disorders | 34 (0.6) | 21 (0.1) | <0.001 |
| Depressive disorders | 227 (3.7) | 250 (1.8) | <0.001 |
| Suicidal ideation | 51 (0.8) | 67 (0.5) | 0.003 |
| * Tested by Pearson’s χ^2^-test with Yates’ continuity correction. | | | |

| **Table S4**. Prevalence and distribution of psychiatric outcomes divided by sex. | | |
| --- | --- | --- |
|  |  | N (%) |
| Total | ♂  ♀ | 6636 (47.1)  7468 (52.9 ) |
| No psychiatric outcome | ♂  ♀ | 6410 (96.6)  7107 (95.2) |
| Any psychiatric outcome | ♂  ♀ | 226 (3.4)  361 (4.8) |
| Alcohol and drug misuse disorders | ♂  ♀ | 82 (1.2)  95 (1.3) |
| Anxiety disorders | ♂  ♀ | 98 (1.5)  185 (2.5) |
| Bipolar/psychotic disorders | ♂  ♀ | 14 (0.2)  7 (0.1) |
| Depressive disorders | ♂  ♀ | 77 (1.2)  173 (2.3) |
| Suicidal ideation | ♂  ♀ | 14 (0.2)  53 (0.7) |

| **Table S5**. Associations between anxiety category at baseline and psychiatric outcome in self-report and parental report, estimated from within-twin analyses conducted on monozygotic twins in the Swedish data. Figures are hazard ratios (95% confidence intervals). | | | | | | | | |
| --- | --- | --- | --- | --- | --- | --- | --- | --- |
|  |  | Anxiety category | | | | | | |
|  |  | Unadjusted | | |  | Adjusted* | | |
| Psychiatric outcome |  | Normal† | Borderline | Abnormal |  | Normal† | Borderline | Abnormal |
| **Self-report** |  |  |  |  |  |  |  |  |
| Any psychiatric outcome |  | 1 | 3.44 (0.66–17.83) | 1.41 (0.59–3.40) |  | 1 | 3.75 (0.70–20.14) | 1.44 (0.58–3.58) |
| Alcohol and drug misuse disorders |  | 1 | NA | 1.00 (0.20–4.96) |  | 1 | NA | 1.39 (0.22–8.68) |
| Anxiety disorders |  | 1 | 4.38 (0.43–44.37) | 1.16 (0.35–3.85) |  | 1 | 5.98 (0.47–75.27) | 1.43 (0.39–5.15) |
| Bipolar/psychotic disorders |  | 1 | NA | NA |  | 1 | NA | NA |
| Depressive disorders |  | 1 | 3.54 (0.35–35.28) | 1.81 (0.64–5.09) |  | 1 | 3.51 (0.35–35.04) | 1.77 (0.60–5.22) |
| Suicidal ideation |  | 1 | NA | NA |  | 1 | NA | NA |
| **Parental report** |  |  |  |  |  |  |  |  |
| Any psychiatric outcome |  | 1 | 1.28 (0.45–3.63) | 1.79 (0.69–4.61) |  | 1 | 0.79 (0.23–2.79) | 3.10 (0.94–10.22) |
| Alcohol and drug misuse disorders |  | 1 | 0.88 (0.13–5.72) | 5.89 (0.70–49.68) |  | 1 | NA | NA |
| Anxiety disorders |  | 1 | 1.46 (0.26–8.09) | 3.21 (0.83–12.39) |  | 1 | 0.66 (0.07–5.84) | 10.11 (1.04–98.03) |
| Bipolar/psychotic disorders |  | 1 | NA | NA |  | 1 | NA | NA |
| Depressive disorders |  | 1 | 1.65 (0.35–7.78) | 4.49 (1.27–15.89) |  | 1 | 1.26 (0.23–6.95) | 5.74 (1.22–26.89) |
| Suicidal ideation |  | 1 | NA | 0.50 (0.05–5.51) |  | 1 | NA | 1.00 (0.06–15.99) |
| * Adjusted for attention-deficit/hyperactivity disorder, autism spectrum disorder, and developmental coordination disorder at CATSS-9/12.  † Reference category. | | | | | | | | |

| **Table S6**. E-values for the associations between anxiety category at baseline and psychiatric outcome in self-report and parental report. Estimates in brackets indicate E-values for the corresponding confidence intervals. | | | | | | | |
| --- | --- | --- | --- | --- | --- | --- | --- |
|  | Anxiety category | | | | | | |
|  | Unadjusted | | |  | Adjusted* | | |
| Psychiatric outcome | Normal† | Borderline | Abnormal |  | Normal† | Borderline | Abnormal |
| **Self-report** |  |  |  |  |  |  |  |
| Any psychiatric outcome | 1 | 3.21 (2.06) | 6.9 (5.31) |  | 1 | 2.85 (1.69) | 6.26 (4.6) |
| Alcohol and drug misuse disorders | 1 | 2.24 (1) | 2.19 (1) |  | 1 | 1.74 (1) | 1.81 (1) |
| Anxiety disorders | 1 | 6.16 (3.78) | 10.96 (7.77) |  | 1 | 4.87 (2.69) | 9.31 (6.12) |
| Bipolar/psychotic disorders | 1 | 2.6 (1) | 1.64 (1) |  | 1 | 3.21 (1) | 2.45 (1) |
| Depressive disorders | 1 | 3.48 (1.64) | 11.76 (8.25) |  | 1 | 2.54 (1) | 9.05 (5.91) |
| Suicidal ideation | 1 | 2.75 (1) | 7.32 (3.52) |  | 1 | 1.59 (1) | 4.09 (1.24) |
| **Parental report** |  |  |  |  |  |  |  |
| Any psychiatric outcome | 1 | 4.64 (3.17) | 9.23 (7.02) |  | 1 | 4.07 (2.66) | 7.22 (5.17) |
| Alcohol and drug misuse disorders | 1 | 2.47 (1) | 5.15 (3) |  | 1 | 2.45 (1) | 3.72 (1.71) |
| Anxiety disorders | 1 | 6.66 (4.03) | 14.52 (10.19) |  | 1 | 5.67 (3.25) | 11.5 (7.56) |
| Bipolar/psychotic disorders | 1 | 3.31 (1) | 2.56 (1) |  | 1 | 2.83 (1) | 1.37 (1) |
| Depressive disorders | 1 | 7.5 (4.58) | 12.52 (8.33) |  | 1 | 6.1 (3.52) | 9.61 (5.85) |
| Suicidal ideation | 1 | 2.52 (1) | 9.23 (4.17) |  | 1 | 2.6 (1) | 6.98 (2.62) |
| * Adjusted for sex, attention-deficit/hyperactivity disorder, autism spectrum disorder, and developmental coordination disorder at age 9/12.  † Reference category. | | | | | | | |

| **Table S7.** Prevalence and distribution of psychiatric outcomes at follow up among ‘normal’, ‘borderline’ and ‘abnormal’ anxiety categories in the Netherlands Twin Register (NTR). | | | | |
| --- | --- | --- | --- | --- |
|  |  | Anxiety category | | |
|  | Total | Normal | Borderline | Abnormal |
|  | 4283* (100) | 3188 (74.4) | 652 (15.2) | 443 (10.3) |
| No psychiatric outcome | 3388 (79.1) | 2780 (87.2) | 423 (64.9) | 185 (41.8) |
| Any psychiatric outcome | 895 (20.9) | 408 (12.8) | 229 (35.1) | 258 (58.2) |
| High-risk/harmful alcohol use | 7 (0.8) | NA | NA | NA |
| Anxiety | 397 (11.1) | 118 (4.4) | 116 (21.3) | 163 (44.7) |
| Thought problems | 393 (14.9) | 200 (10.4) | 86 (20.0) | 107 (37.7) |
| Depression | 391 (10.9) | 144 (5.4) | 103 (18.9) | 144 (39.5) |
| Suicidal ideation | 59 (2.4) | 21 (1.2) | 13 (3.2) | 25 (9.2) |
| * N = individuals were data was eligible for any psychiatric outcome. | | | | |

| **Table S8.** Associations between anxiety category at baseline and psychiatric outcome in the Netherlands Twin Register (NTR). Figures are odds ratios (95% confidence intervals). | | | | | | | |
| --- | --- | --- | --- | --- | --- | --- | --- |
|  | Anxiety category | | | | | | |
|  | Unadjusted | | |  | Adjusted* | | |
| Psychiatric outcome | Normal† | Borderline | Abnormal |  | Normal† | Borderline | Abnormal |
| Any psychiatric outcome | 1 | **3.69 (3.01–4.45)** | **9.50 (7.60–11.89)** |  | 1 | **3.15 (2.58–3.84)** | **7.53 (5.94–9.55)** |
| High-risk/harmful alcohol use | 1 | 0.70 (0.08–5.85) | 0.00 (0.00–0.00) |  | 1 | 1.44 (0.22–9.48) | 0.00 (0.00–0.00) |
| Anxiety | 1 | **5.83 (4.44–7.64)** | **17.40 (13.12–23.08)** |  | 1 | **5.20 (3.77–6.68)** | **14.02 (10.38–18.93)** |
| Thought problems | 1 | **2.15 (1.63–2.82)** | **5.21 (3.91–6.93)** |  | 1 | **1.92 (1.44–2.57)** | **4.29 (3.15–5.83)** |
| Depression | 1 | **4.08 (3.11–5.34)** | **11.40 (8.63–15.05)** |  | 1 | **3.12 (2.35–4.15)** | **8.00 (5.93–10.79)** |
| Suicidal ideation | 1 | **2.81 (1.40–5.65)** | **8.69 (4.80–15.71)** |  | 1 | 2.30 (1.08–4.88) | **6.59 (3.24–13.40)** |
| * Adjusted for sex, attention-deficit/hyperactivity disorder and autism spectrum disorder at ages 7, 10, and 12. Bold estimates indicate significance at the 0.005 level, except for ‘any psychiatric outcome’, where alpha was set to 0.05. | | | | | | | |

**Figures**

**Figure S1.** Cumulative incidence of alcohol and drug misuse disorders in individuals within 'normal', 'borderline', and 'abnormal' anxiety categories at age 15 in self-report and parental report.

*Note.* For representational purposes, figures were cut at age 21.

**Figure S2.** Cumulative incidence of anxiety disorders in individuals within 'normal', 'borderline', and 'abnormal' anxiety categories at age 15 in self-report and parental report.

*Note.* For representational purposes, figures were cut at age 21.

**Figure S3.** Cumulative incidence of bipolar/psychotic disorders in individuals within 'normal', 'borderline', and 'abnormal' anxiety categories at age 15 in self-report and parental report.

*Note.* For representational purposes, figures were cut at age 21.

**Figure S4.** Cumulative incidence of depressive disorders in individuals within 'normal', 'borderline', and 'abnormal' anxiety categories at age 15 in self-report and parental report.

*Note.* For representational purposes, figures were cut at age 21.

**Figure S5.** Cumulative incidence of suicidal ideation in individuals within 'normal', 'borderline', and 'abnormal' anxiety categories at age 15 in self-report and parental report.

*Note.* For representational purposes, figures were cut at age 21.

**Figure S6.** Attributable fraction of alcohol and drug misuse disorders in individuals within 'borderline' and 'abnormal' anxiety categories at age 15 in self-report and parental report.

*Note.* Dashed lines represent 95% confidence intervals. For representational purposes, figures were cut at age 21.

**Figure S7.** Attributable fraction of anxiety disorders in individuals within 'borderline' and 'abnormal' anxiety categories at age 15 in self−report and parental report.

*Note.* Dashed lines represent 95% confidence intervals. For representational purposes, figures were cut at age 21.

**Figure S8.** Attributable fraction of bipolar/psychotic disorders in individuals within 'borderline' and 'abnormal' anxiety categories at age 15 in self−report and parental report.

*Note.* Dashed lines represent 95% confidence intervals. For representational purposes, figures were cut at age 20.

**Figure S9.** Attributable fraction of depressive disorders in individuals within 'borderline' and 'abnormal' anxiety categories at age 15 in self−report and parental report.

*Note.* Dashed lines represent 95% confidence intervals. For representational purposes, figures were cut at age 21.

**Figure S10.** Attributable fraction of suicidal ideation in individuals within 'borderline' and 'abnormal' anxiety categories at age 15 in self−report and parental report.

*Note.* Dashed lines represent 95% confidence intervals. For representational purposes, figures were cut at age 21.
