# Supplementary figures and images for: Anxiety at age 15 predicts psychiatric diagnoses and suicidal ideation in late adolescence and young adulthood: results from two longitudinal studies
Source: BMC Psychiatry. 2019 Nov 14;19:363. doi: 10.1186/s12888-019-2349-3 (PMC6857289; doi:10.1186/s12888-019-2349-3)

### Self-report

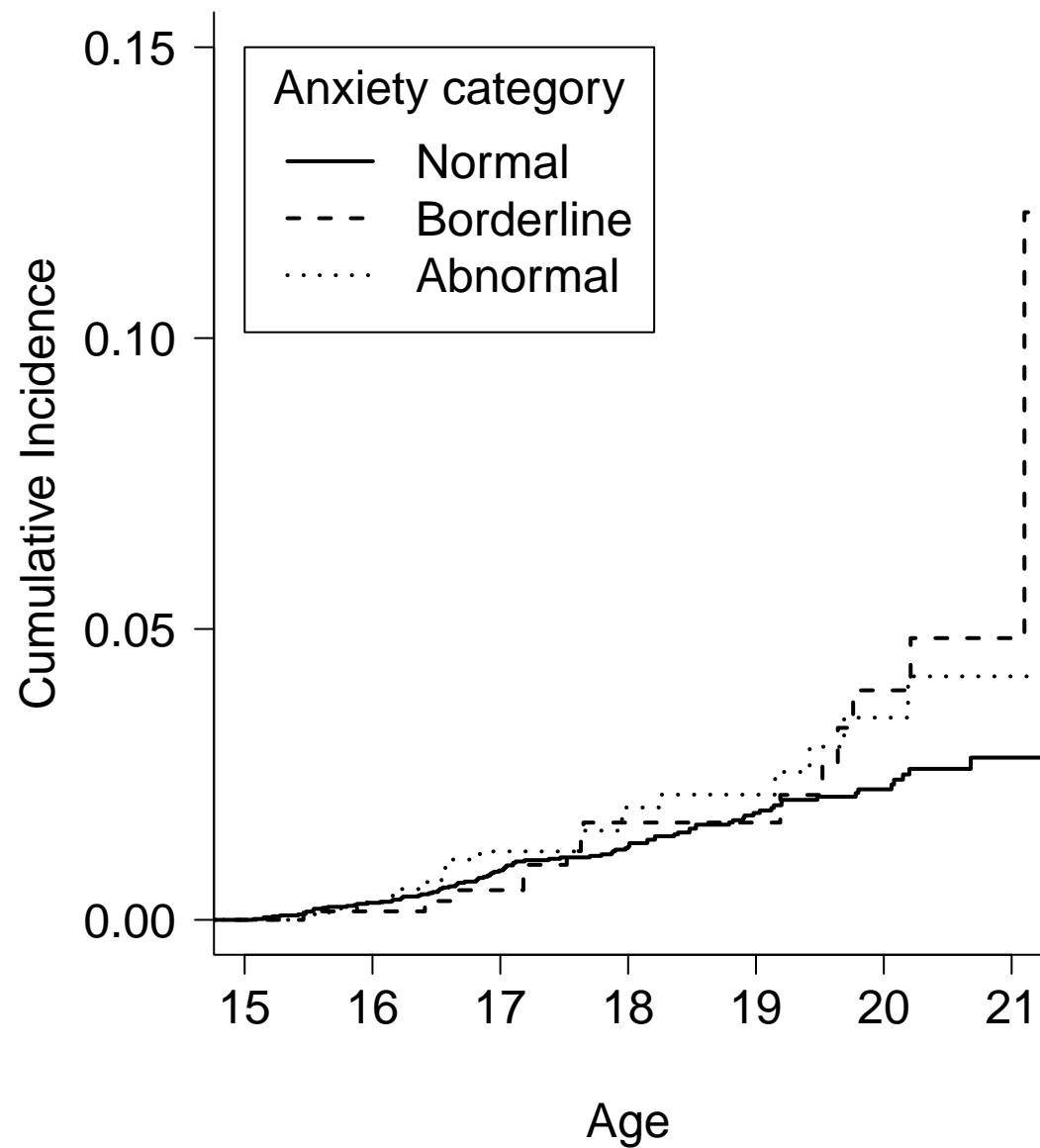

### Parental report

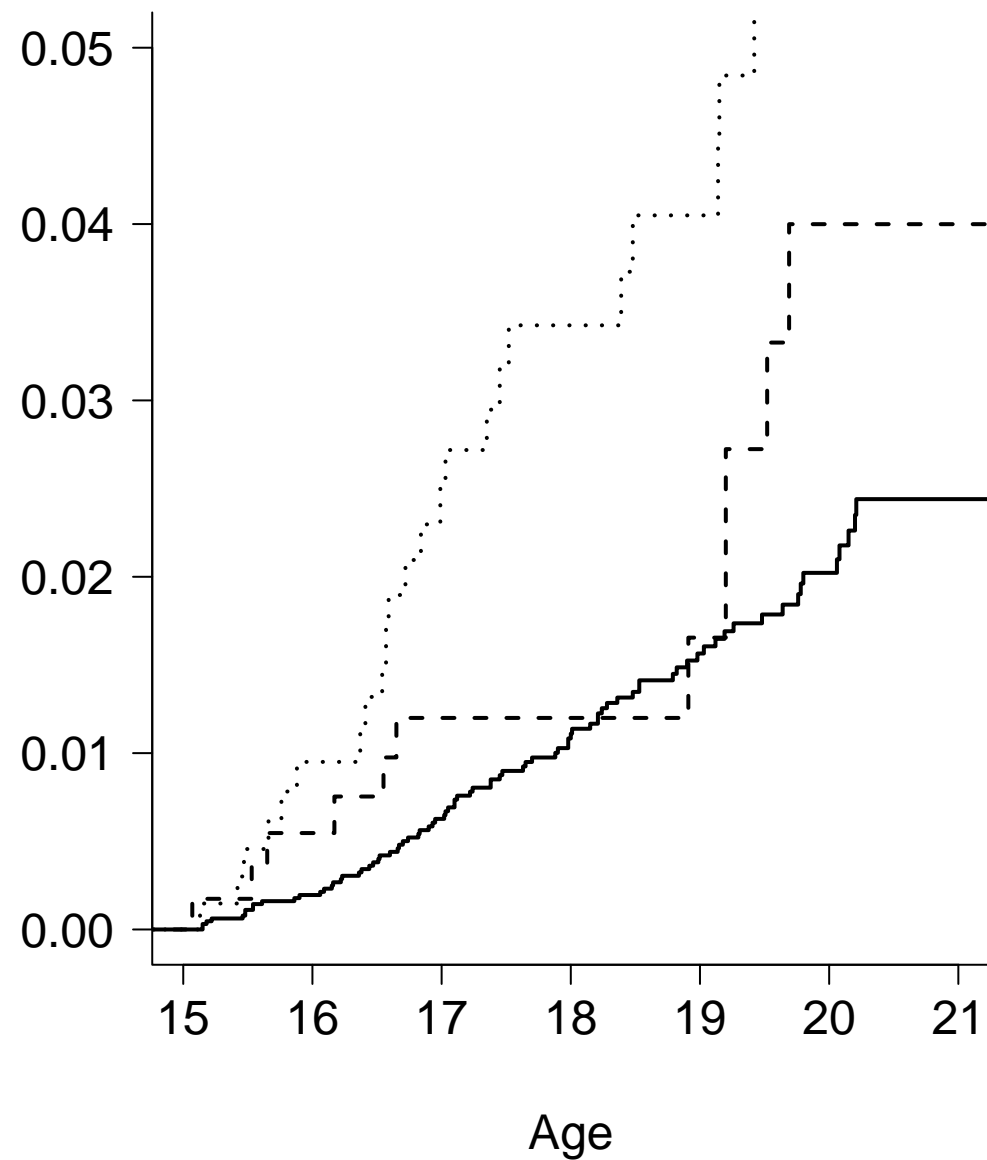

Supplement: Supplementary file 2 — Additional file 2: Figure S1. Cumulative incidence of alcohol and drug misuse disorders in individuals within 'normal', 'borderline', and 'abnormal' anxiety categories at age 15 in self-report and parental report. Note. For representational purposes, figures were cut at age 21. [file 12888_2019_2349_MOESM2_ESM.pdf]

### Self-report

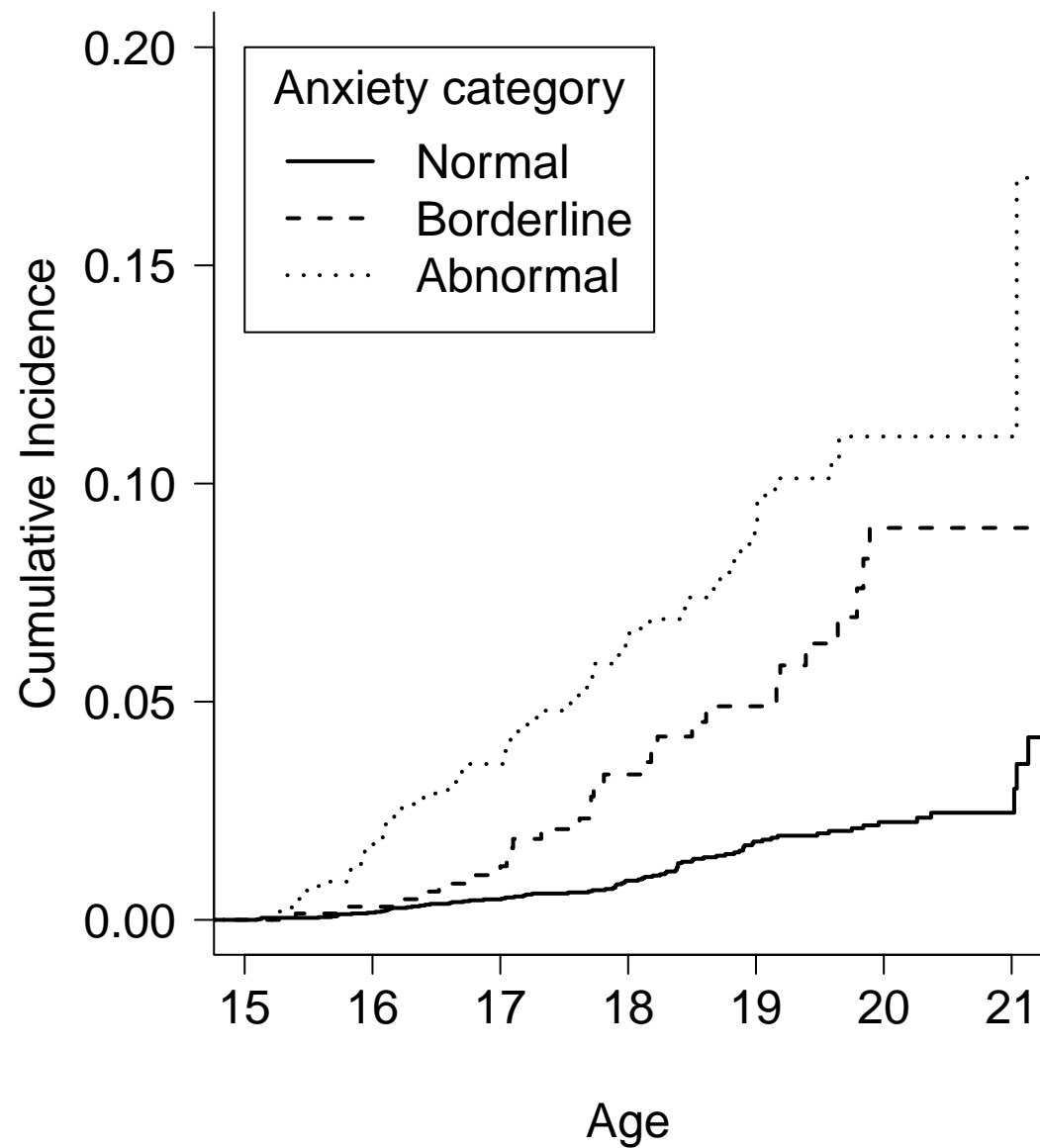

### Parental report

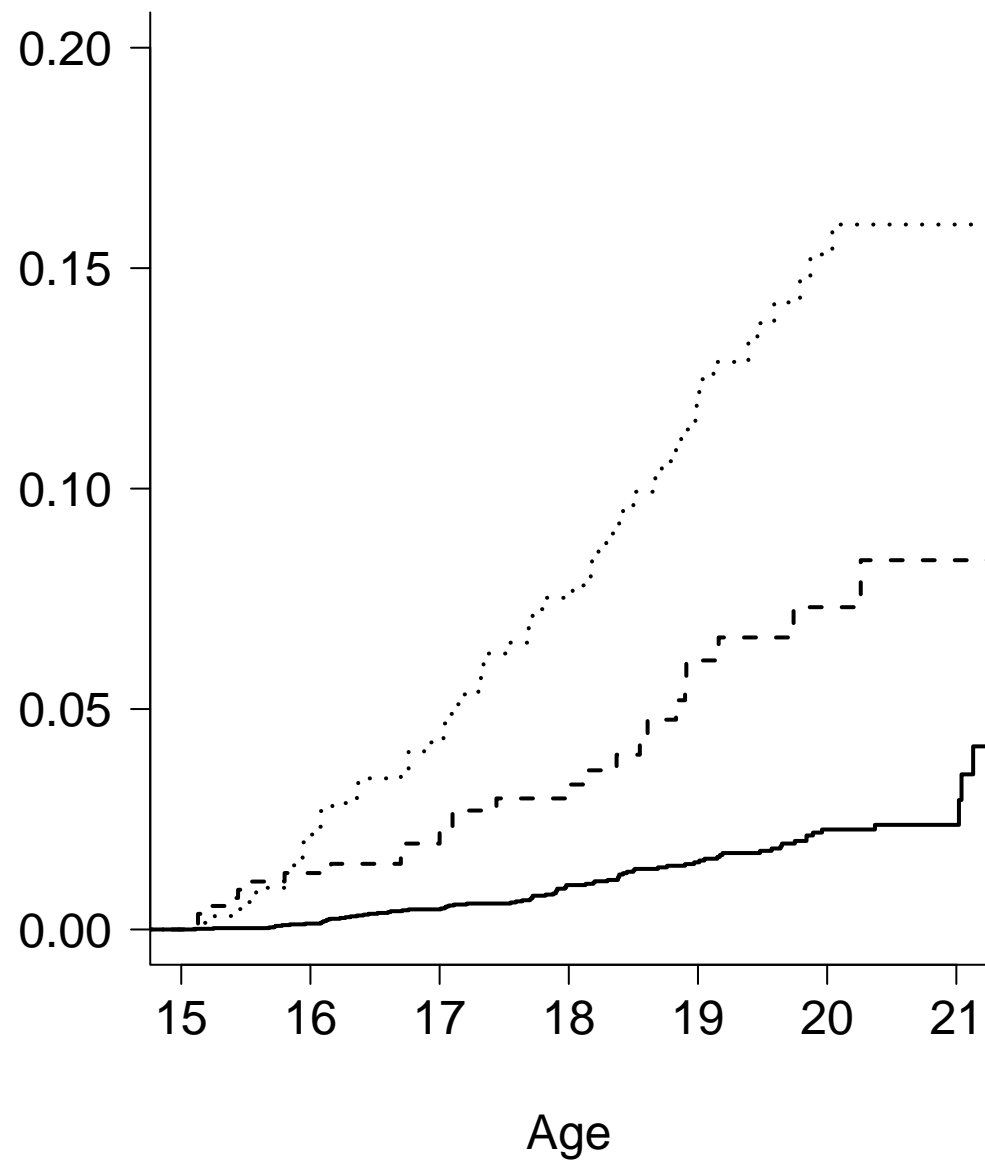

Supplement: Supplementary file 3 — Additional file 3: Figure S2. Cumulative incidence of anxiety disorders in individuals within 'normal', 'borderline', and 'abnormal' anxiety categories at age 15 in self-report and parental report. Note. For representational purposes, figures were cut at age 21. [file 12888_2019_2349_MOESM3_ESM.pdf]

### Self-report

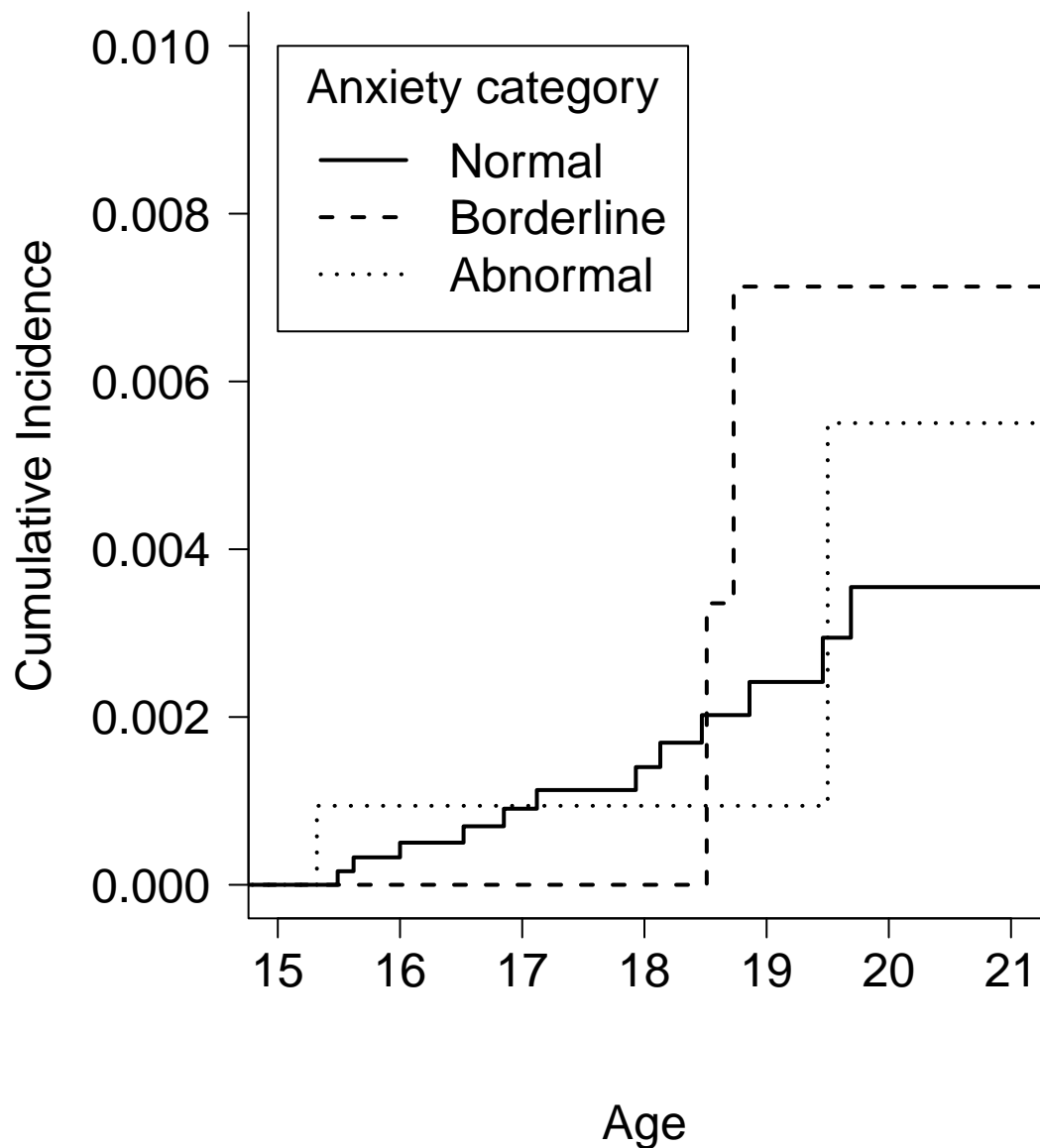

### Parental report

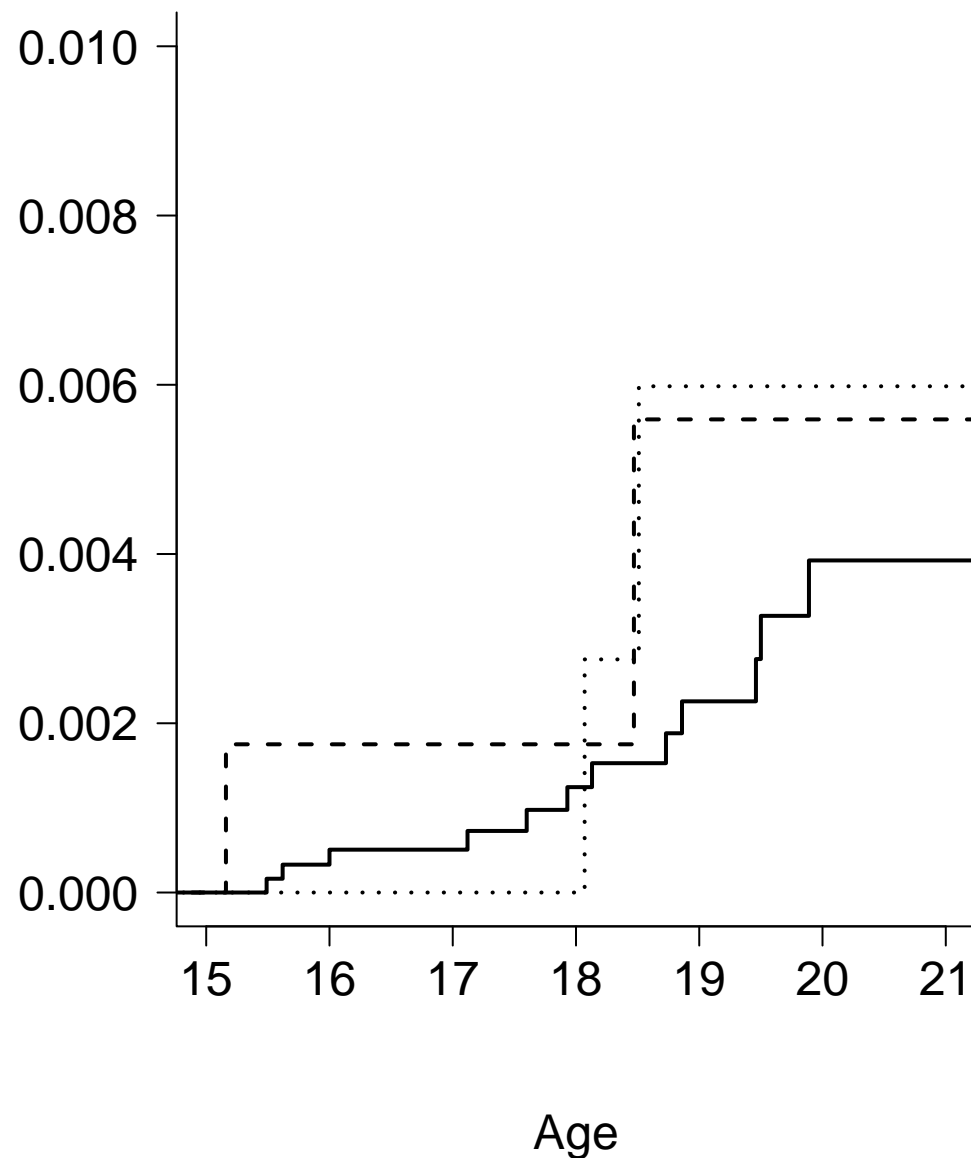

Supplement: Supplementary file 4 — Additional file 4: Figure S3. Cumulative incidence of bipolar/psychotic disorders in individuals within 'normal', 'borderline', and 'abnormal' anxiety categories at age 15 in self-report and parental report. Note. For representational purposes, figures were cut at age 21. [file 12888_2019_2349_MOESM4_ESM.pdf]

### Self-report

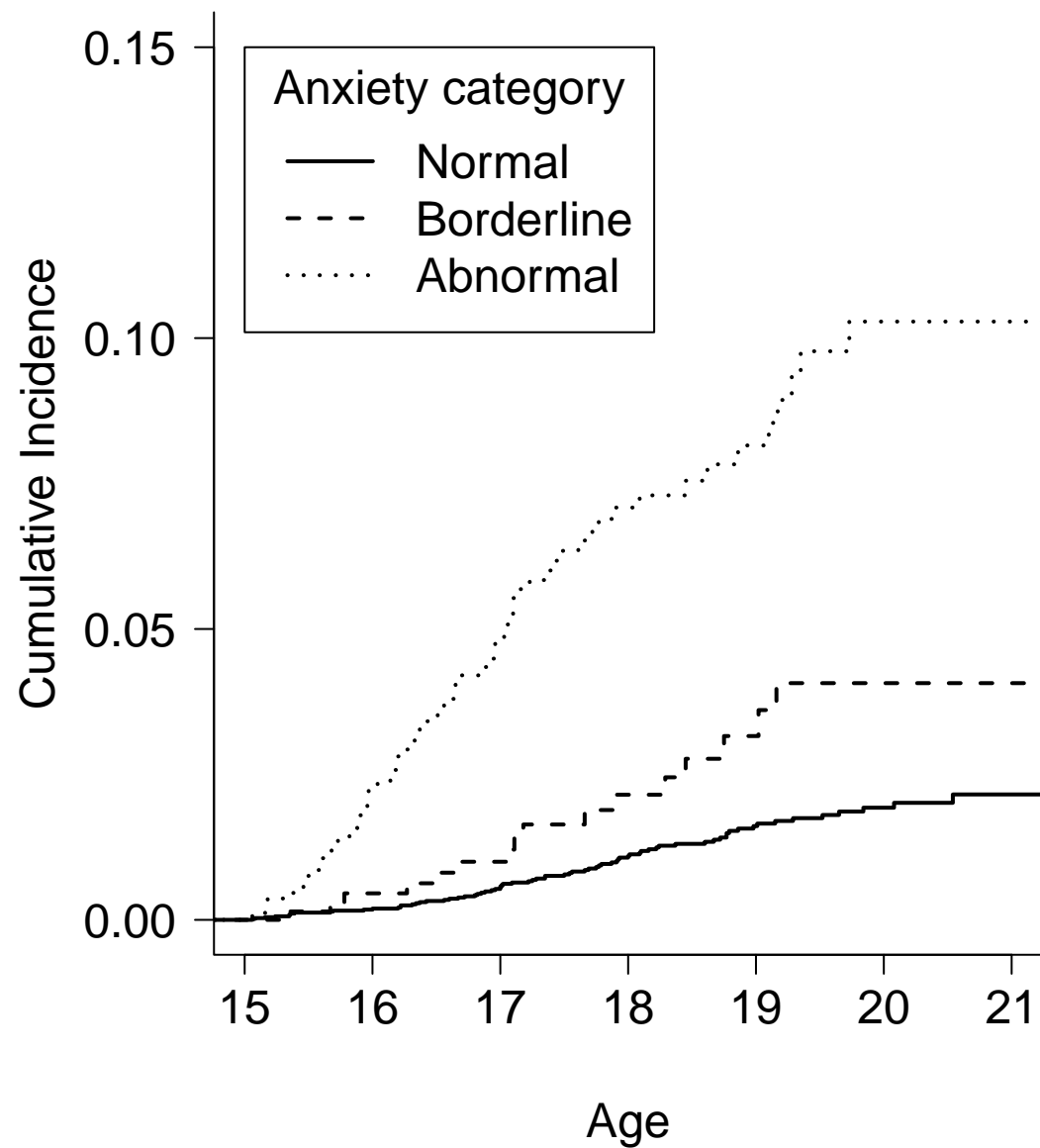

### Parental report

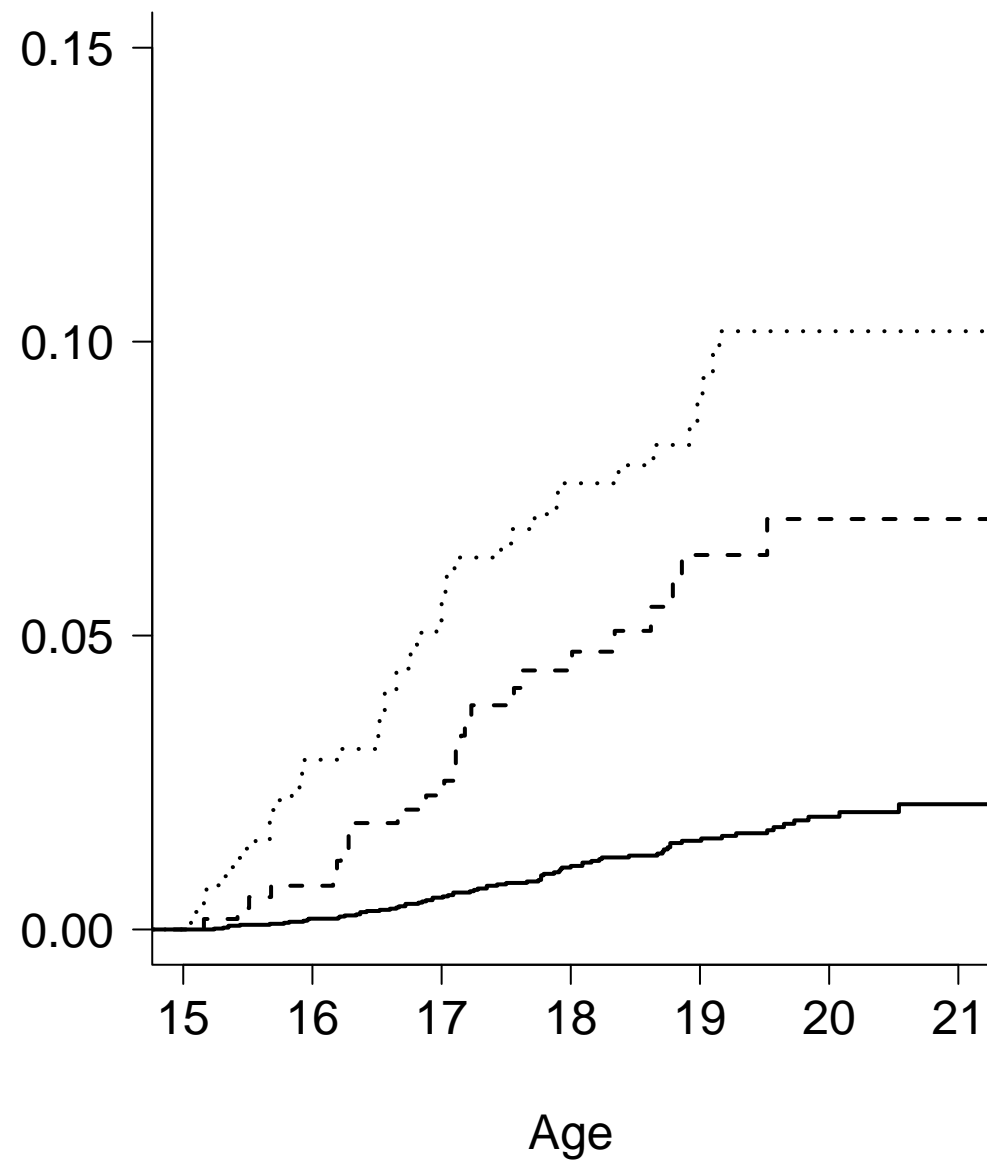

Supplement: Supplementary file 5 — Additional file 5: Figure S4. Cumulative incidence of depressive disorders in individuals within 'normal', 'borderline', and 'abnormal' anxiety categories at age 15 in self-report and parental report. Note. For representational purposes, figures were cut at age 21. [file 12888_2019_2349_MOESM5_ESM.pdf]

### Self-report

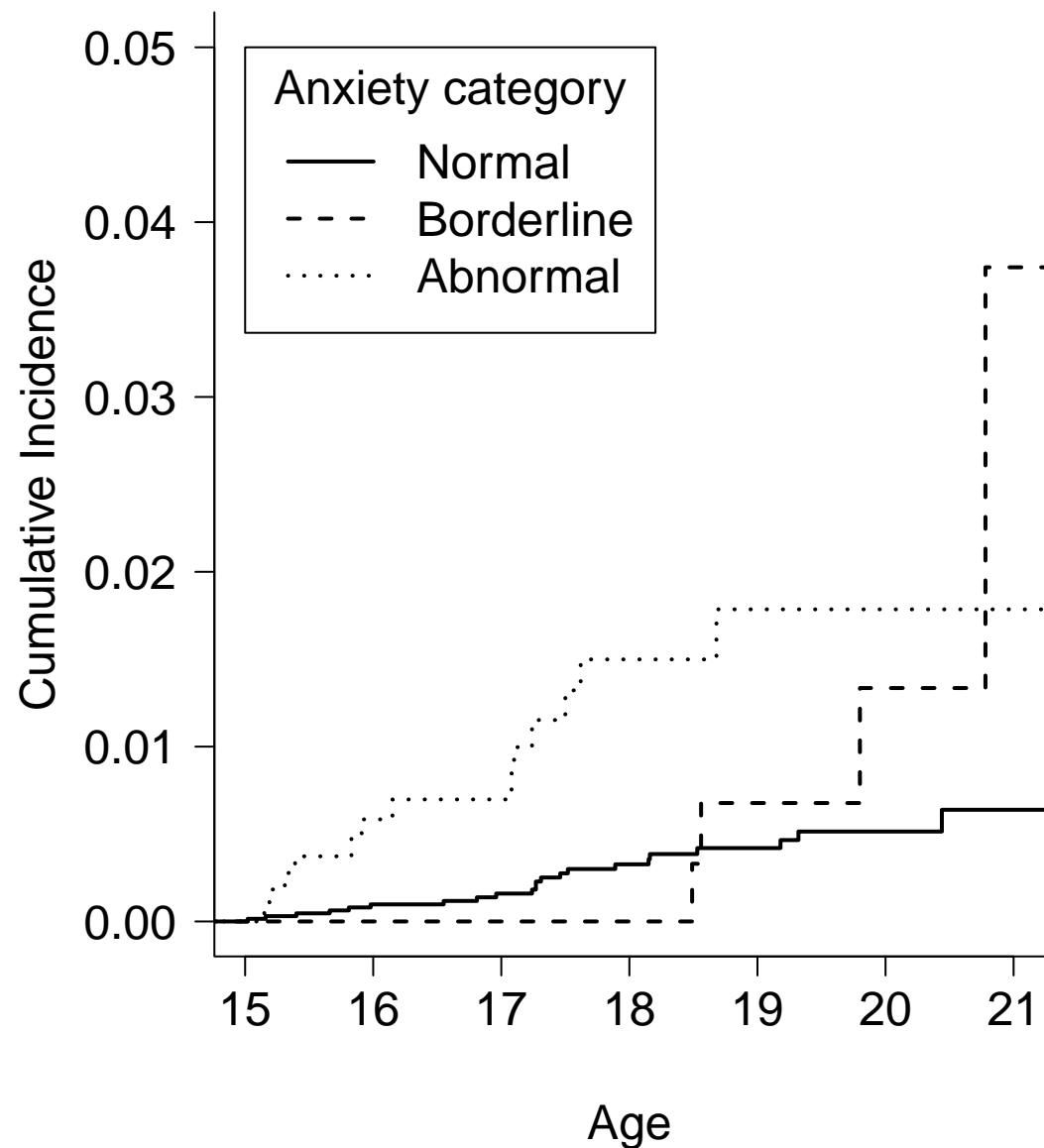

### Parental report

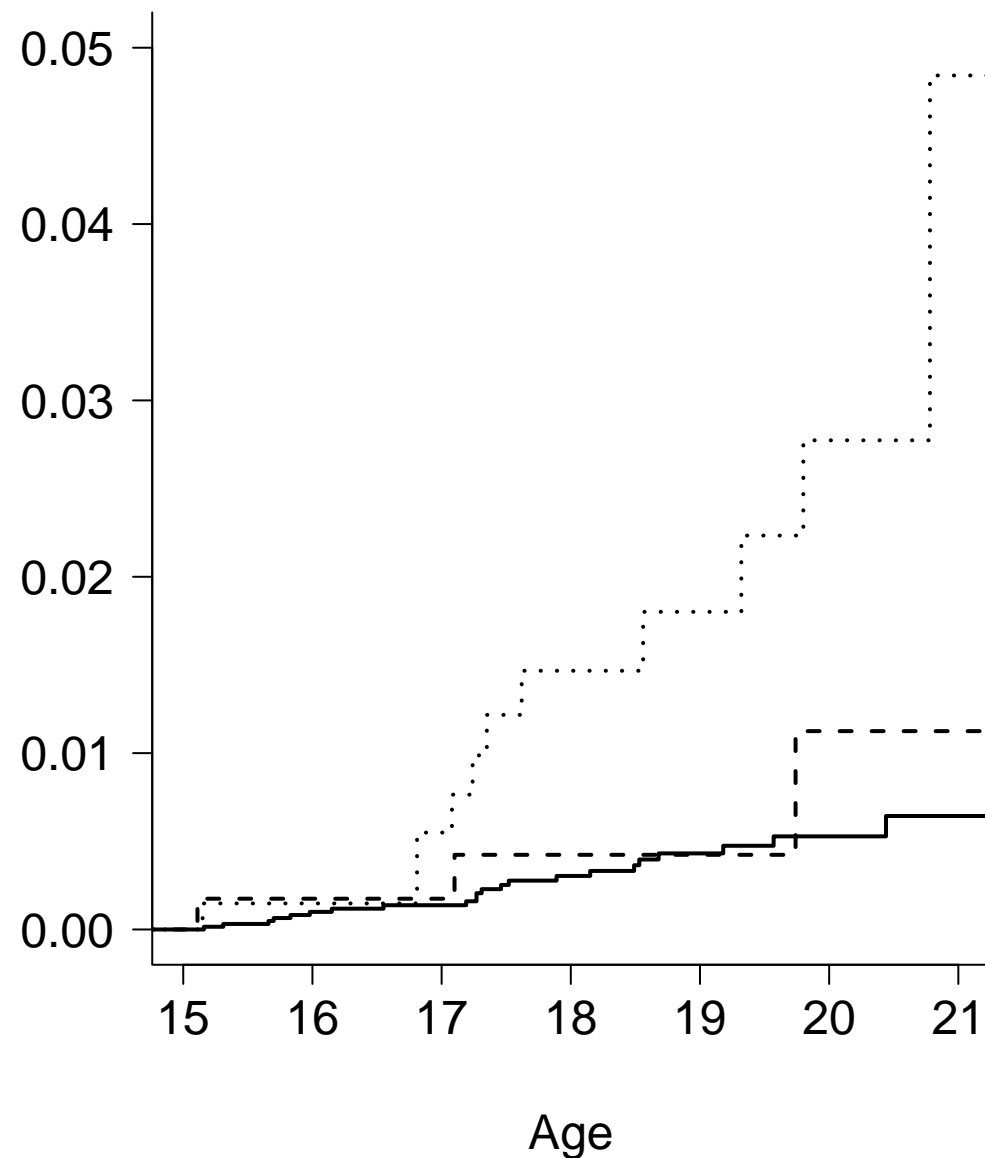

Supplement: Supplementary file 6 — Additional file 6: Figure S5. Cumulative incidence of suicidal ideation in individuals within 'normal', 'borderline', and 'abnormal' anxiety categories at age 15 in self-report and parental report. Note. For representational purposes, figures were cut at age 21. [file 12888_2019_2349_MOESM6_ESM.pdf]

**Self-report**

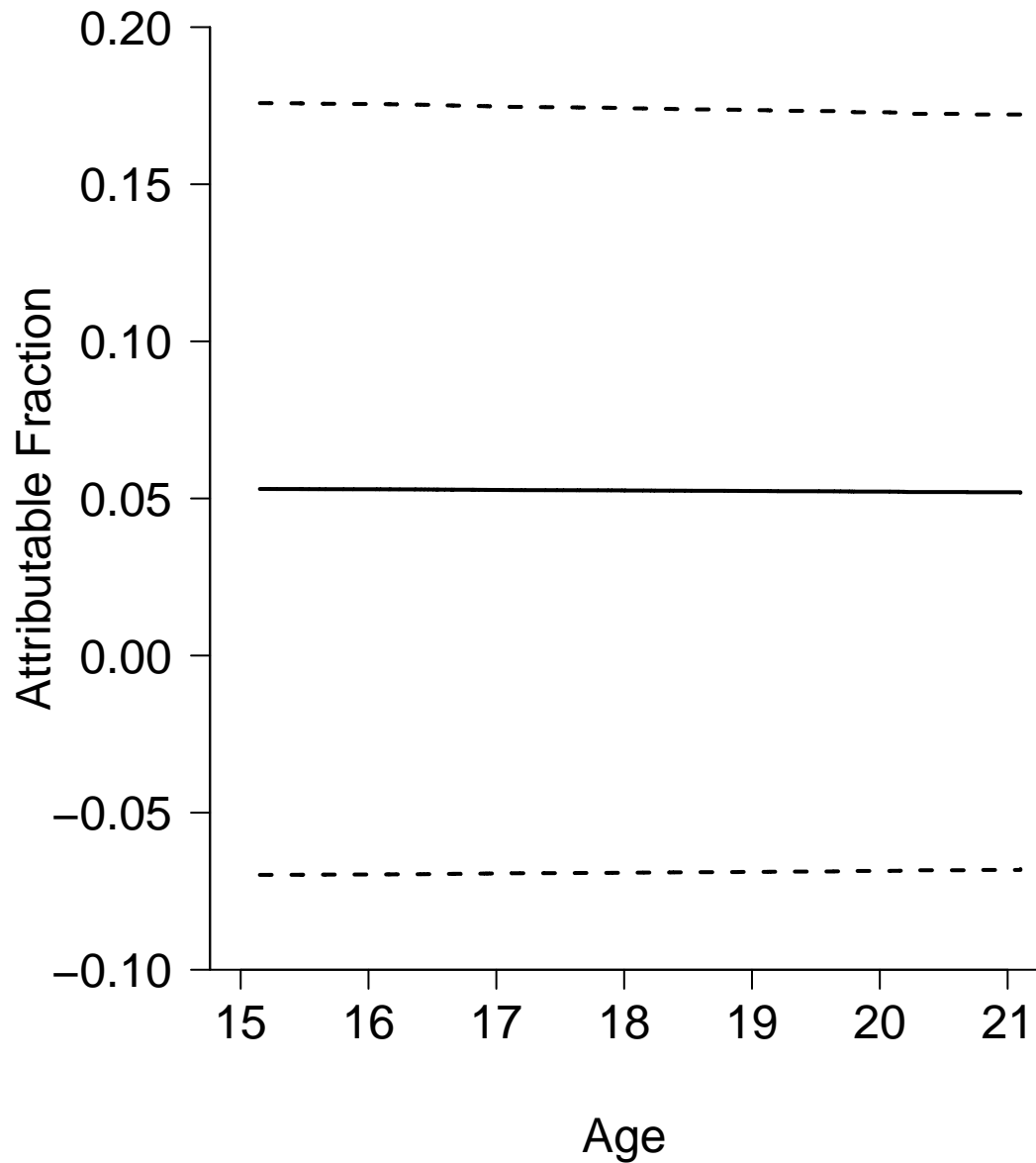

**Parental report**

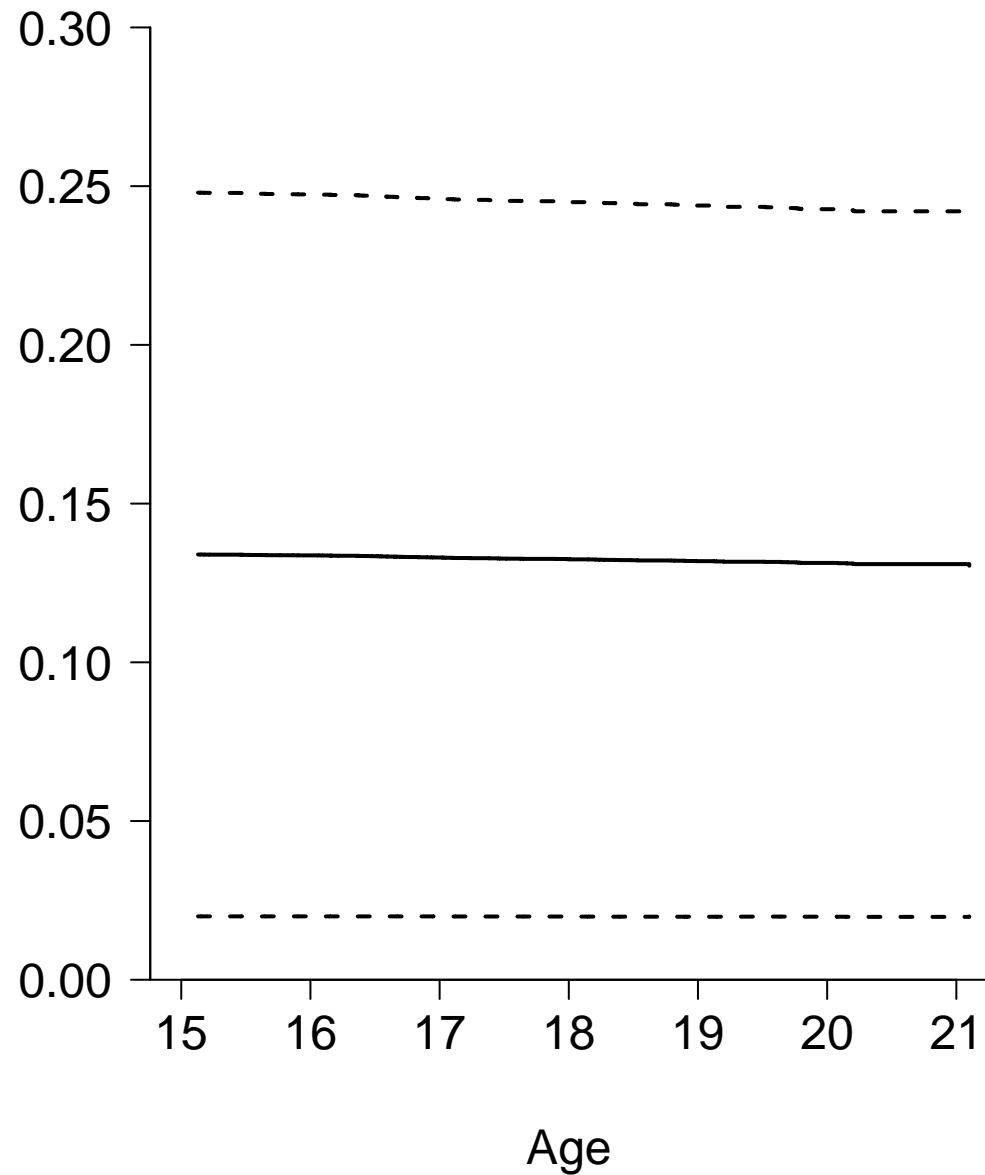

Supplement: Supplementary file 7 — Additional file 7: Figure S6. Attributable fraction of alcohol and drug misuse disorders in individuals within 'borderline' and 'abnormal' anxiety categories at age 15 in self-report and parental report. Note. Dashed lines represent 95% confidence intervals. For representational purposes, figures were cut at age 21. [file 12888_2019_2349_MOESM7_ESM.pdf]

**Self-report**

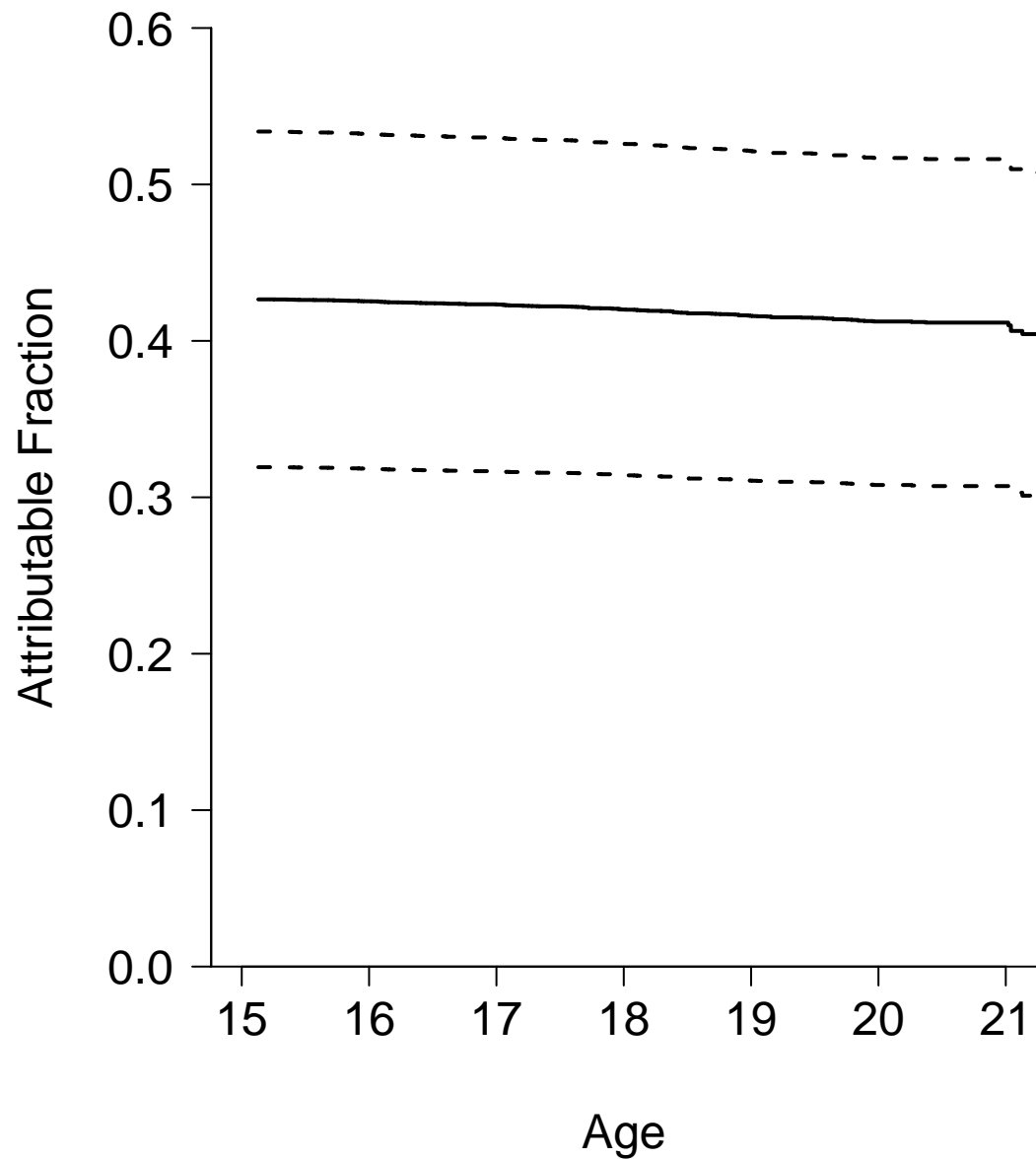

**Parental report**

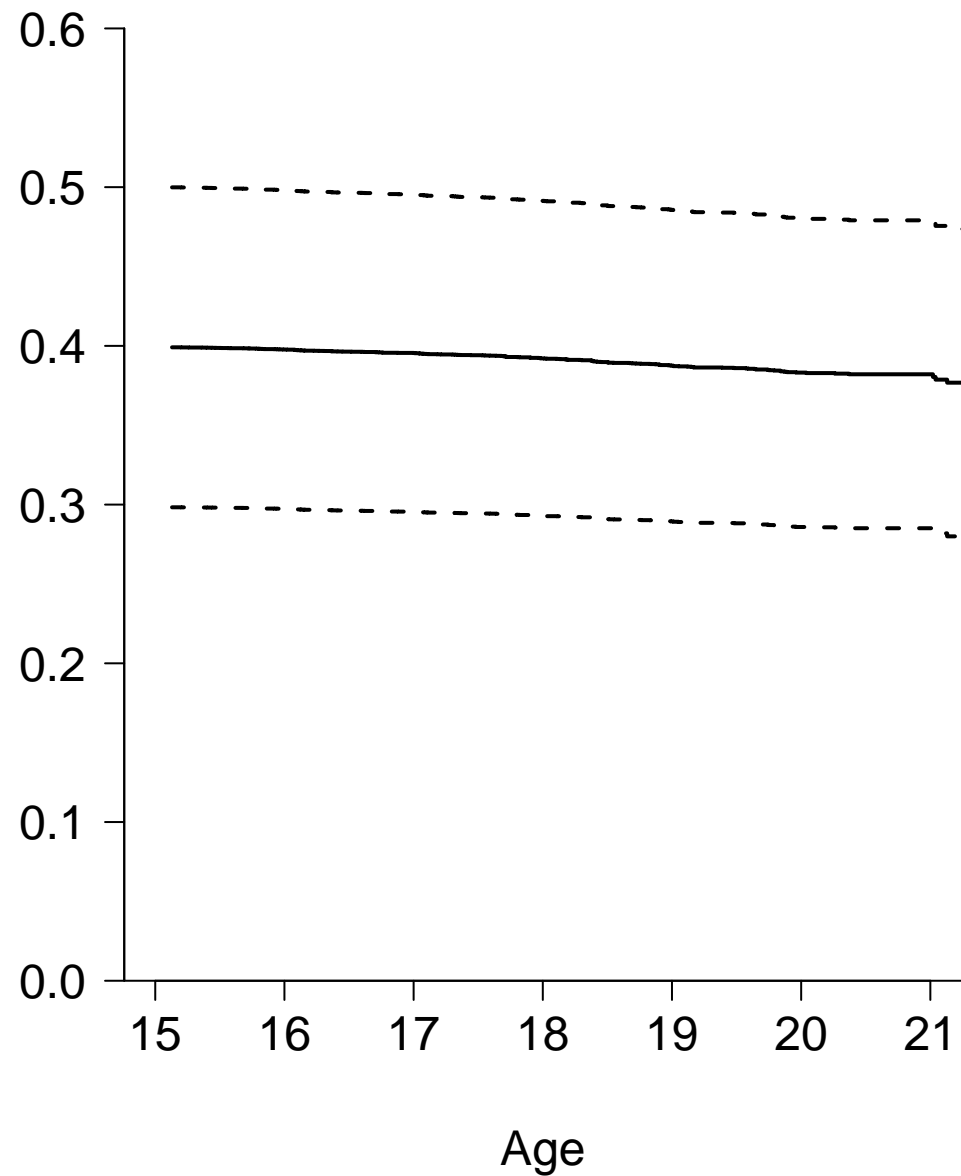

Supplement: Supplementary file 8 — Additional file 8: Figure S7. Attributable fraction of anxiety disorders in individuals within 'borderline' and 'abnormal' anxiety categories at age 15 in self−report and parental report. Note. Dashed lines represent 95% confidence intervals. For representational purposes, figures were cut at age 21. [file 12888_2019_2349_MOESM8_ESM.pdf]

**Self-report**

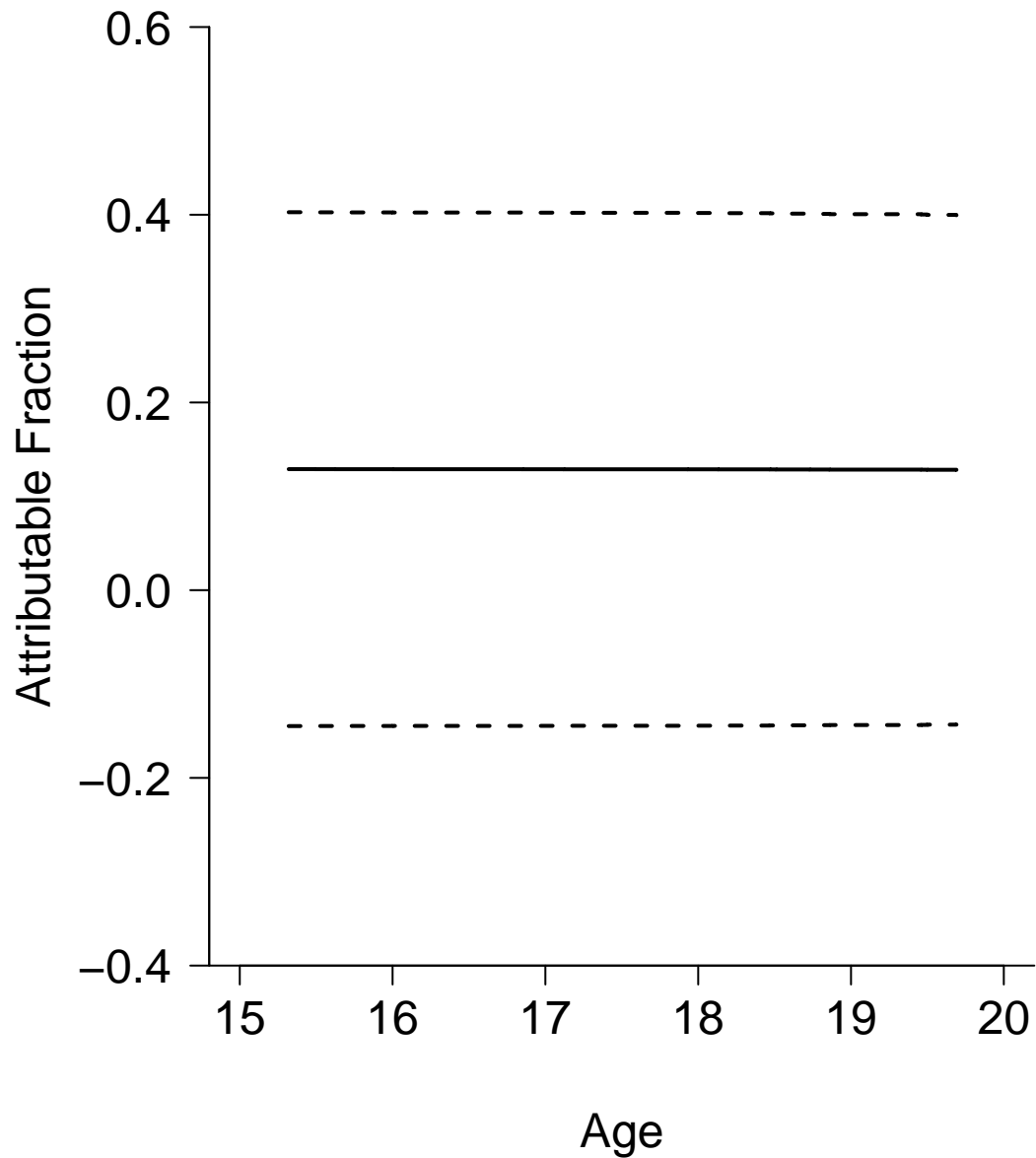

**Parental report**

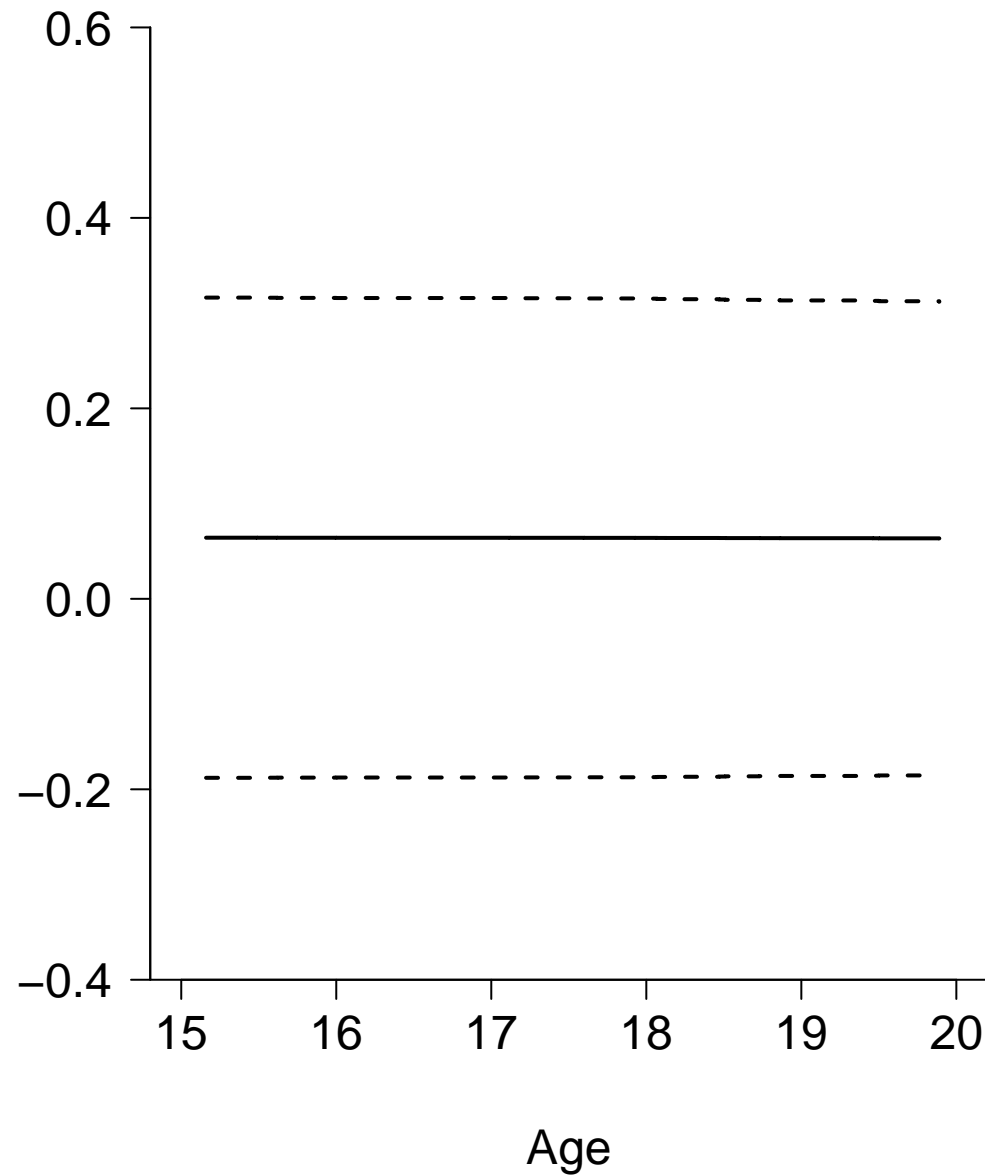

Supplement: Supplementary file 9 — Additional file 9: Figure S8. Attributable fraction of bipolar/psychotic disorders in individuals within 'borderline' and 'abnormal' anxiety categories at age 15 in self−report and parental report. Note. Dashed lines represent 95% confidence intervals. For representational purposes, figures were cut at age 20. [file 12888_2019_2349_MOESM9_ESM.pdf]

**Self-report**

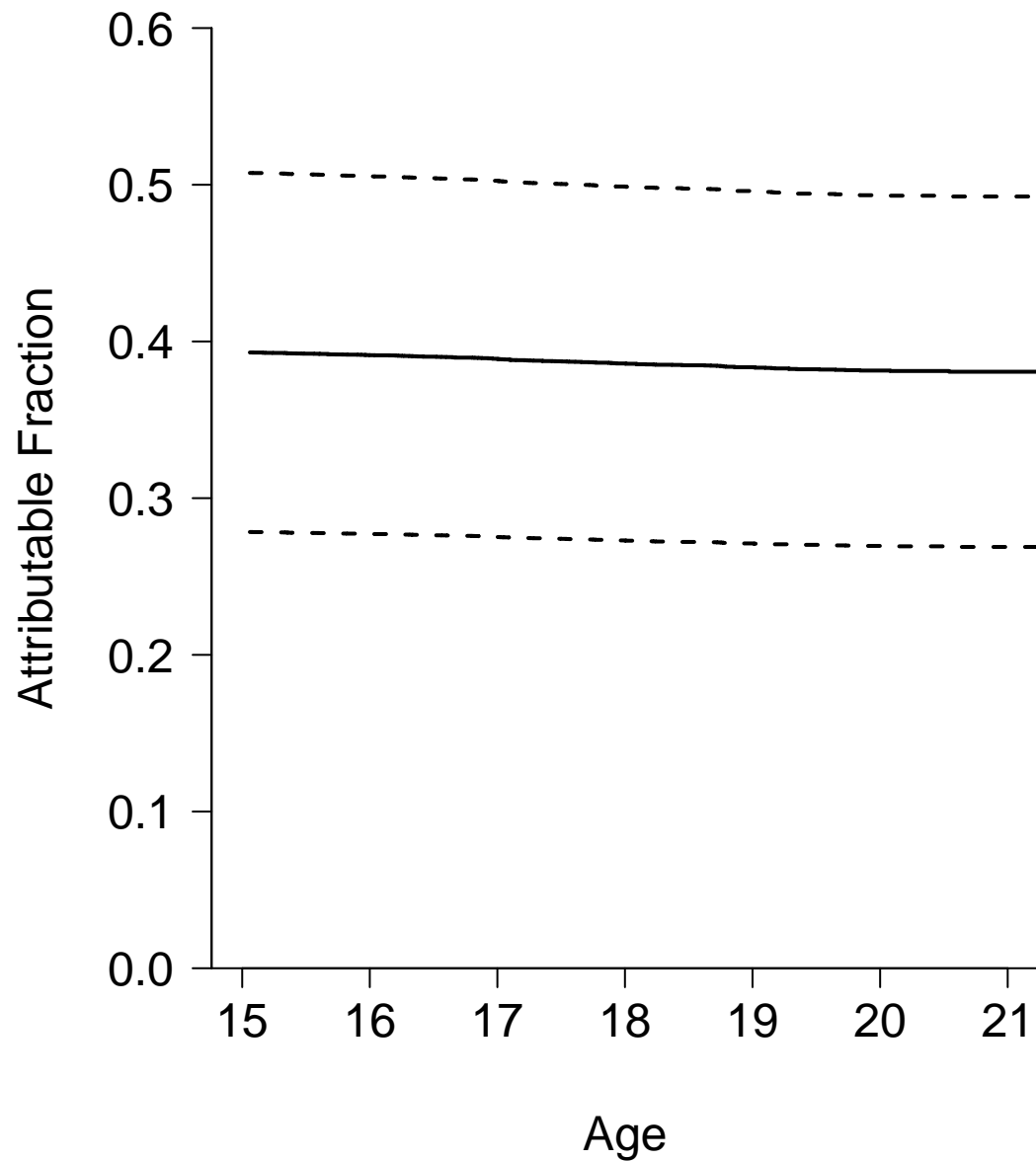

**Parental report**

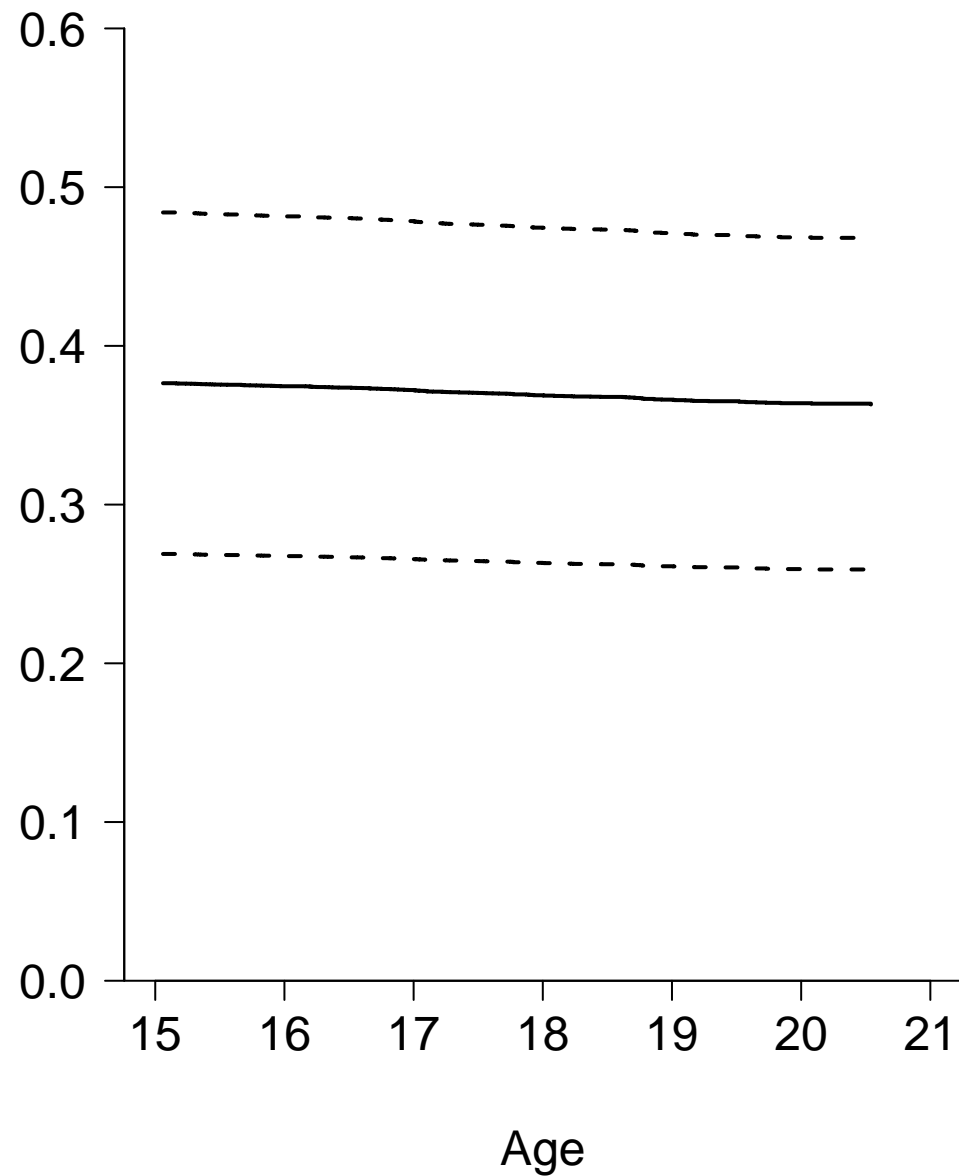

Supplement: Supplementary file 10 — Additional file 10: Figure S9. Attributable fraction of depressive disorders in individuals within 'borderline' and 'abnormal' anxiety categories at age 15 in self−report and parental report. Note. Dashed lines represent 95% confidence intervals. For representational purposes, figures were cut at age 21. [file 12888_2019_2349_MOESM10_ESM.pdf]

**Self-report**

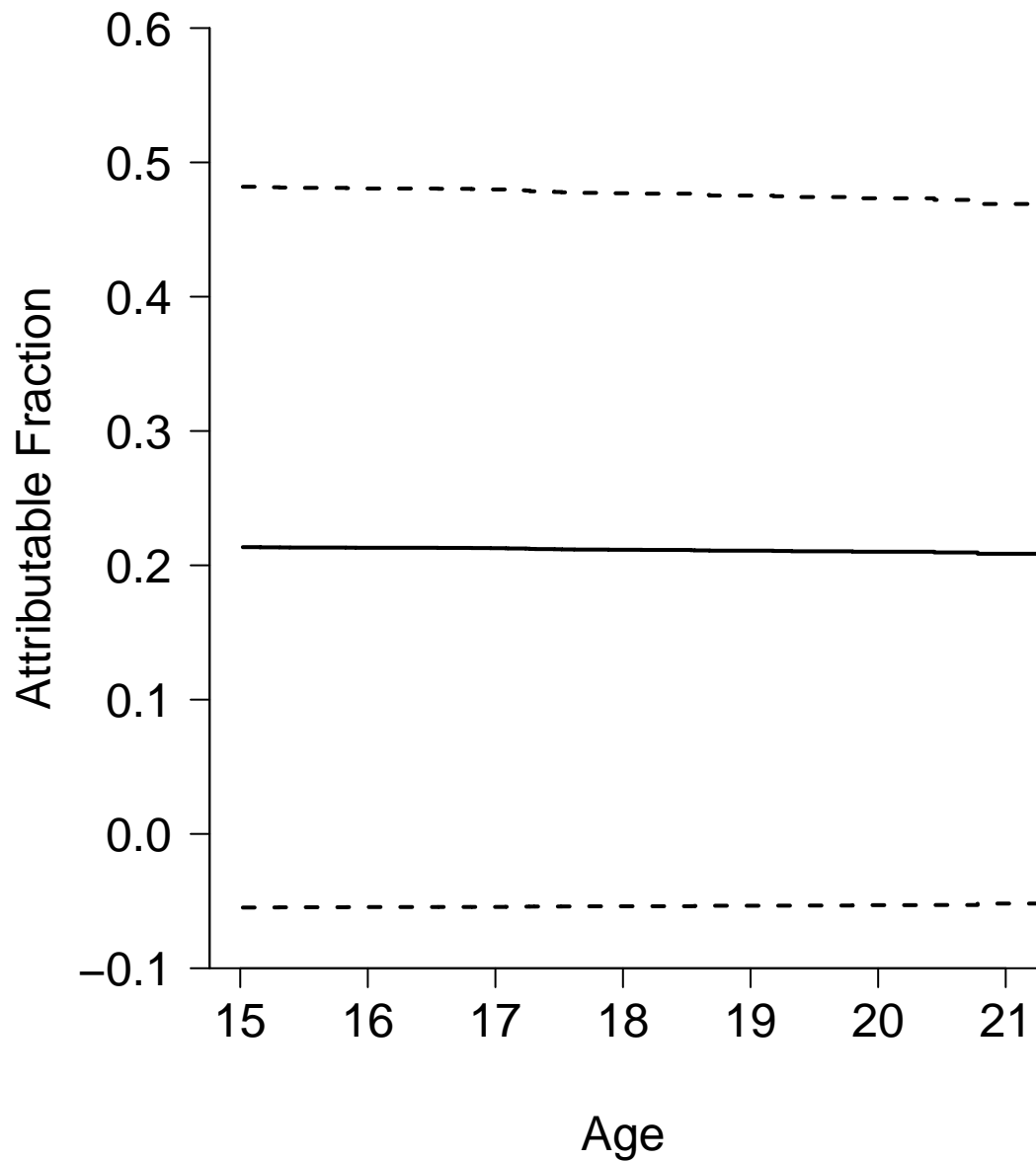

**Parental report**

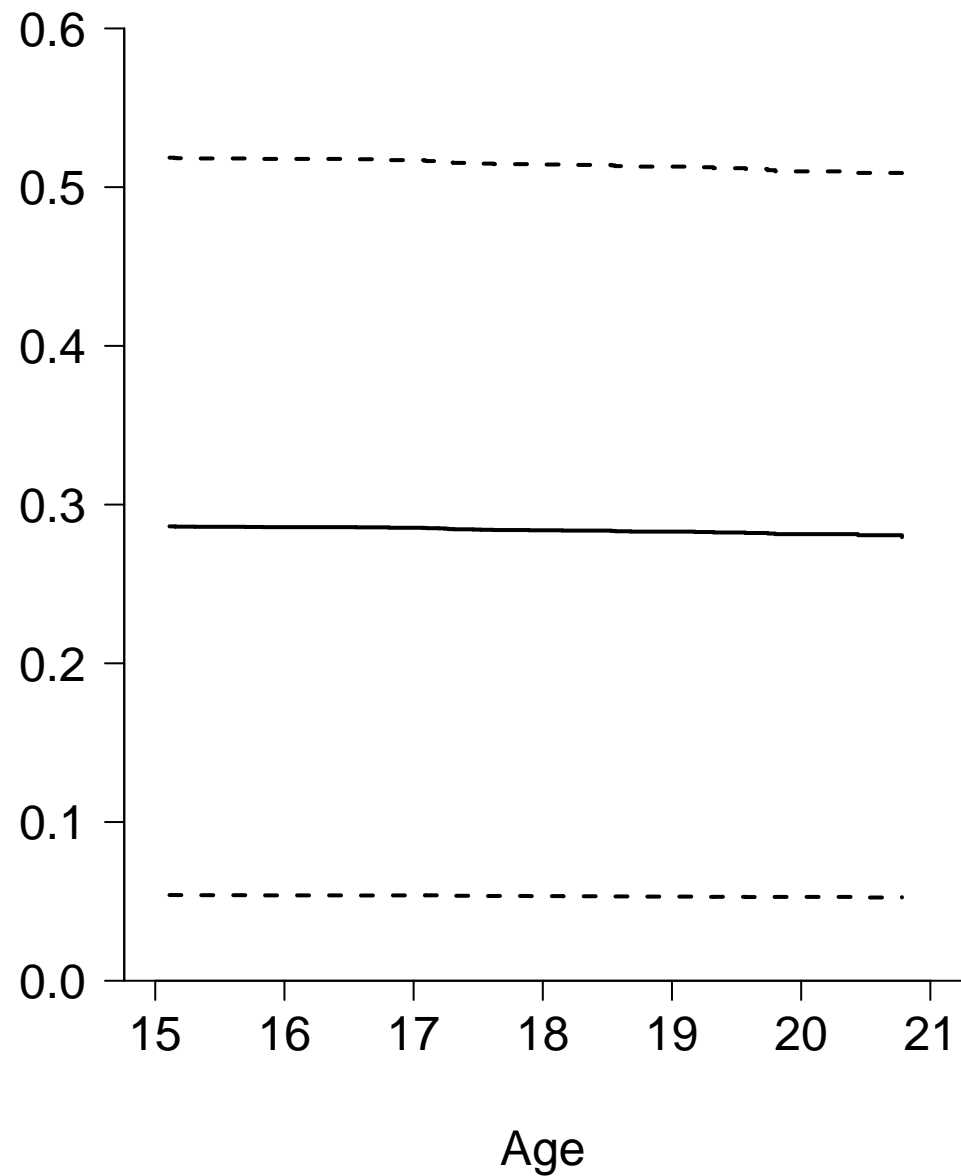

Supplement: Supplementary file 11 — Additional file 11: Figure S10. Attributable fraction of suicidal ideation in individuals within 'borderline' and 'abnormal' anxiety categories at age 15 in self−report and parental report. Note. Dashed lines represent 95% confidence intervals. For representational purposes, figures were cut at age 21. [file 12888_2019_2349_MOESM11_ESM.pdf]
